# Molecular footprinting of skeletal tissues in the catshark *Scyliorhinus canicula* and the clawed frog *Xenopus tropicalis* identifies conserved and derived features of vertebrate calcification

**DOI:** 10.3389/fgene.2015.00283

**Published:** 2015-09-15

**Authors:** Sébastien Enault, David N. Muñoz, Willian T. A. F. Silva, Véronique Borday-Birraux, Morgane Bonade, Silvan Oulion, Stéphanie Ventéo, Sylvain Marcellini, Mélanie Debiais-Thibaud

**Affiliations:** ^1^Institut des Sciences de l'Evolution de Montpellier, UMR5554, Université Montpellier, Centre National de la Recherche Scientifique, IRD, EPHEMontpellier, France; ^2^Laboratory of Development and Evolution, Department of Cell Biology, Faculty of Biological Sciences, Universidad de ConcepciónConcepción, Chile; ^3^Laboratoire EGCE UMR Centre National de la Recherche Scientifique 9191, IRD247, Université Paris SudGif-sur-Yvette, France; ^4^Université Paris Diderot, Sorbonne Paris CitéParis, France; ^5^Institute for Neurosciences of Montpellier, Institut National de la Santé et de la Recherche Médicale U1051Montpellier, France

**Keywords:** fibrillar collagens, vertebrate skeletogenesis, bone, cartilage, *Scyliorhinus canicula*, *Xenopus tropicalis*

## Abstract

Understanding the evolutionary emergence and subsequent diversification of the vertebrate skeleton requires a comprehensive view of the diverse skeletal cell types found in distinct developmental contexts, tissues, and species. To date, our knowledge of the molecular nature of the shark calcified extracellular matrix, and its relationships with osteichthyan skeletal tissues, remain scarce. Here, based on specific combinations of expression patterns of the *Col1a1, Col1a2*, and *Col2a1* fibrillar collagen genes, we compare the molecular footprint of endoskeletal elements from the chondrichthyan *Scyliorhinus canicula* and the tetrapod *Xenopus tropicalis*. We find that, depending on the anatomical location, *Scyliorhinus* skeletal calcification is associated to cell types expressing different subsets of fibrillar collagen genes, such as high levels of *Col1a1* and *Col1a2* in the neural arches, high levels of *Col2a1* in the tesserae, or associated to a drastic *Col2a1* downregulation in the centrum. We detect low *Col2a1* levels in *Xenopus* osteoblasts, thereby revealing that the osteoblastic expression of this gene was significantly reduced in the tetrapod lineage. Finally, we uncover a striking parallel, from a molecular and histological perspective, between the vertebral cartilage calcification of both species and discuss the evolutionary origin of endochondral ossification.

## Introduction

The evolutionary origin and diversification of the skeleton remains one of the most intriguing issue in vertebrate biology. Solving this problem requires a comprehensive view of the diversity of skeletal cell types found in distinct developmental contexts, tissues, anatomical locations, and species, as has been emphasized in a recent synthesis of existing skeletal terminologies (Dahdul et al., 2012). In mammals, the chondrocytes produce the extracellular matrix of the fibrous, elastic and hyaline cartilage, while osteoblasts and osteocytes are involved in bone formation (Benjamin and Evans, 1990; Hartmann, 2009; Zhang et al., 2009; Long, 2011). Yet, an intermediate type of chondroid bone, exhibiting characteristics of both bone and cartilage, has been described in rodents as well as teleosts, leading some authors to propose that, in fact, bone and cartilage represent two extreme forms of a skeletal tissue continuum (Huysseune and Verraes, 1986; Huysseune and Sire, 1990; Mizoguchi et al., 1997; Kranenbarg et al., 2005; Estêvão et al., 2011). In addition, chondrichthyans display a series of heavily calcified skeletal tissues such as the cartilaginous tesserae of the jaws (with no obvious homologs in osteichthyans, see Dean et al., 2005; Dean and Summers, 2006; Dean et al., 2009; Omelon et al., 2014), the vertebral body developing around the notochord (Peignoux-Deville et al., 1982; Dean and Summers, 2006; Eames et al., 2007; Porter et al., 2007; Fleming et al., 2015) and the perichondrium of the neural arches laying on each side of the neural tube (Peignoux-Deville et al., 1982; Eames et al., 2007). In summary, while developmental and paleontological studies have revealed the versatile nature of skeletal tissues characterizing the vertebrate skeleton (Donoghue and Sansom, 2002; Janvier and Arsenault, 2002; Dahdul et al., 2012; Janvier, 2015), the molecular identity and the evolutionary relationships of the distinct vertebrate skeletal cell types remain an open question.

The comparison of expression patterns represents a powerful approach to examine cell type evolution and, for instance, has shed light on the origin of sensory neurons in animals (Arendt, 2008). Here, we have explored the possibility that combinations of expression patterns of genes coding for crucial components of the skeletal matrix can serve as useful molecular footprints to compare the identity of skeletal cell types between chondrichthyan and osteichthyan representatives. We chose to focus on the *Col1a1, Col1a2*, and *Col2a1* genes, belonging to the Clade A of the fibrillar collagen family, because they are known to contribute to biomineralization and because they are intimately associated to skeletal development and evolution (Wada et al., 2006; Rychel and Swalla, 2007; Zhang and Cohn, 2008; Landis and Silver, 2009; Eyre and Weis, 2013; Veis and Dorvee, 2013). *Col1a1* and *Col1a2* (Type I collagen) are robustly expressed in osteichthyan osteoblasts (Kobayashi and Kronenberg, 2005; Li et al., 2009; Albertson et al., 2010; Estêvão et al., 2011; Eames et al., 2012). By contrast, the osteoblastic expression of *Col2a1* (Type II collagen) is more variable and has been reported in developing bones of gar and teleosts (Benjamin and Ralphs, 1991; Albertson et al., 2010; Eames et al., 2012), at low levels in some scattered mouse osteoblasts (Hilton et al., 2007), and transiently in chick preosteoblasts (Abzhanov et al., 2007). In addition, *Col2a1* displays a conserved expression pattern in chondrocytes of immature hyaline cartilage whose proliferation drives the growth of endochondral bones (Benjamin and Ralphs, 1991; Nah et al., 2001; Kerney and Hanken, 2008; Hartmann, 2009; Albertson et al., 2010; Estêvão et al., 2011; Eames et al., 2012). *Col2a1* expression becomes progressively downregulated as the hyaline cartilage matures and calcifies its extracellular matrix (Eames et al., 2003; Hartmann, 2009). Of particular relevance for this study, *Col2a1*-negative mature cartilage calcification usually occurs at levels that are too weak to robustly stain with Alizarin red, a reagent commonly used to specifically detect the calcifying bone matrix of vertebrate embryos (Kirsch et al., 1997; Khanarian et al., 2014), with some exceptions reported in the swell shark vertebrae and the domestic fowl trachea (Hogg, 1982; Eames et al., 2007). Possibly due to lineage-specific duplications, lamprey and hagfish (cyclostomes) exhibit one or two *Col2a1* orthologs (and no *Col1a1* or *Col1a2* genes) expressed in broad regions encompassing mesenchymal cells and some, but not all, cartilaginous elements (Zhang and Cohn, 2006, 2008; Zhang et al., 2006; Ota and Kuratani, 2010; Cattell et al., 2011). In shark, immunohistochemistry allowed the clear detection of Type II collagen fibers in cartilage matrix, while the weaker reactivity of the anti-Type I collagen antibody suggested a perichondral expression, without allowing the discrimination of cells secreting Col1a1 and/or Col1a2 proteins (Eames et al., 2007).

In order to identify skeletal cell types sharing a specific molecular identity between remotely related jawed vertebrates, we compared the endoskeletal expression patterns of the *Col1a1, Col1a2*, and *Col2a1* fibrillar collagen genes in the chondrichthyan *Scyliorhinus canicula* (*S.c.*) and the tetrapod *Xenopus tropicalis* (*X.t.*). We find that, depending on the anatomical location, skeletal calcification in *S.c.* occurs in the vicinity of cell types expressing distinct combinations of fibrillar collagen genes. In particular, calcification is associated to perichondral cells expressing high levels of *Col1a1* and *Col1a2* in the neural arches, and to chondrocytes expressing high levels of *Col2a1* in the tesserae or experiencing a drastic *Col2a1* downregulation in the centrum. In *X.t.*, the moderate expression of *Col2a1* in some osteoblasts differs from the situation described in actinopterygians and amniotes, suggesting that the osteoblastic expression of this gene was significantly reduced in the tetrapod lineage. Finally, we observe a striking parallel between the internal calcification of the vertebral cartilage of *X.t.* and *S.c.* and discuss the evolutionary origins of endochondral ossification.

## Materials and methods

### *Scyliorhinus canicula* biological material

*Scyliorhinus canicula* embryos were obtained at the Station Méditerrannéenne de l'Environnement Littoral (SMEL, Sète, France) and raised in the laboratory at 18°C. Embryos were euthanized by overdose of MS-222 (Sigma) following all animal-care specifications of the European ethics legislation. Whole embryos were fixed 48 h in PFA 4% in PBS 1 × at 4°C and then transferred in ethanol at −20°C for storage. Dissected body parts (jaws or trunk sections) were rehydrated and transferred to a 25% sucrose bath for cryosection at 14 μm thickness, and stored at −20°C on alternative slides to get comparable sections on each slide. These sections were used for *in situ* hybridizations and Alizarin red—Alcian blue histological staining (see following sections). Dissected body parts were decalcified in MORSE (sodium citrate 10% and formic acid 20%) solution for 5 days before being transferred to paraplast blocs and sectioned at 10 μm thickness. These sections were used for Hematoxylin-Eosin-Safran (HES) histological staining and anti-Col2 immunofluorescence. To perform immunofluorescence, dissected trunk slices from 6.7 cm-long embryos and dissected jaw from 9 cm-long embryo were demineralized for 3 h in MORSE solution at room temperature prior to dehydratation, embedded in paraplast and cut at 10–12 μm thickness.

### Histological and immunological stainings

The same histological procedures were performed for the catshark and clawed frog samples. Histological Alizarin red/Alcian blue double staining was performed by rehydrating samples 1 min in phosphate-buffered saline (PBS) 1X, incubating 30 s in a 0.005% Alizarin red S solution (in KOH 0.5%), washing once with PBS 1X, incubating for 2 min in a 0.02% Alcian blue 8G in solution (in 8:2 ethanol/glacial acid acetic), and washing once in EtOH 100% and once in PBS 1X. The slides were then mounted in mowiol. Hematoxilin-Eosin-Safran (HES) histological staining was performed following standard protocols. Col2 immunofluorescence was performed using a 1/200 dilution of a primary anti-collagen II (II-II6B3; Developmental Studies Hybridoma Bank, Iowa City, IA, USA) and a 1/1500 dilution of a secondary Goat polyclonal anti-Mouse IgG—AlexaFluor 594 (Abcam ab150116). For epitope retrieval, sections were treated with trypsin 0.05% (Sigma) in 0.1% CaCl2 buffer at pH7.8 buffer during 10 min at 37°C. Cell nuclei were counterstained with Hoechst.

### *Scyliorhinus canicula* collagen clones

Plasmids containing partial or complete collagen cDNA sequences were obtained through screening of a cDNA library of embryo RNA extracts (Oulion et al., 2010). Specific clones were identified by BLAST as *Scyliorhinus canicula* (Sc-) *Collagen1a1* gene (Sc-*Col1a1*, NCBI accession numbers EU241868.1 and KT261785), *Collagen1a2* gene (Sc-*Col1a2*, NCBI accession numbers EU241869.1 and KT261784), and *Collagen2a1* gene (Sc-*Col2a1*, NCBI accession number EU241867.1). The sequences and details of the clones are provided in the Data Sheet 1. The phylogenetic relationships between proteic sequences were inferred by using the Maximum Likelihood method based on the Le_Gascuel_2008 model (Le and Gascuel, 2008). Initial tree(s) for the heuristic search were obtained by applying the Neighbor-Joining method to a matrix of pairwise distances estimated using a JTT model. A discrete Gamma distribution was used to model evolutionary rate differences among sites [4 categories (+G, parameter = 0.7935)]. The analysis involved 16 amino acid sequences. All positions containing gaps and missing data were eliminated. There were a total of 544 positions in the final dataset. Evolutionary analyses were conducted in MEGA6 (Tamura et al., 2013).

### *Scyliorhinus canicula* and *Xenopus tropicalis* probes

PCR products from specific amplification on Sc-*Col1a2* and Sc-*Col2a1* cDNA inserts were ligated into the pGEM-Teasy vector using the TA cloning kit (Promega). *Sc-Col1a1* was directly amplified from the original cDNA clone. *Xenopus tropicalis* (Xt-) Xt-*Col1a1* (NM_001011005.1), Xt-*Col1a2* (NM_001079250.1), and Xt-*Col2a1* (NM_203889) were amplified from stage NF60 hindlimb cDNA containing both bone and cartilage and blunt-cloned into the pBluescript vector. The PCR primers used in this study are given in Supplementary Table 1. Antisense DIG riboprobes were synthesized using the DIG RNA labeling mix (Roche) and the T3, T7 or Sp6 RNA polymerase (Promega) following the manufacturer's instructions. DIG-labeled riboprobes were purified on MicroSpin G50 column (GE Healthcare).

### *In situ* hybridization on *Scyliorhinus canicula* sections

DIG-labeled probes were hybridized at 70°C overnight, sections were washed twice in 50% formamide, 1 × SSC, 0.1% Tween-20 for 1 h at 70°C, twice in MABT buffer for 30 min before blocking in blocking buffer (MABT, 2% blocking reagent from Roche, 20% inactivated sheep serum) for 2 h at room temperature. Sections were then exposed overnight to a 1:2000 dilution of anti-DIG-AP conjugate antibody (Roche) at 4°C. After washing, slides were incubated with NBT-BCIP (Roche) staining solution according to the manufacturer's instructions and the reaction stopped by washing in water. Images of *in situ* hybridizations and histological stainings were taken under a Hamamatsu NanoZoomer 2.0-HT Slide Scanner (40 × objective). Sense probe negative *in situ* hybridization results are shown in Data Sheet 2.

### *Xenopus tropicalis* animal care and *in situ* hybridization procedure

Adult frogs are routinely maintained at the University of Concepcion following standard protocols established for *Xenopus tropicalis*. Embryos and tadpoles were obtained by natural mating and staged according to the Nieuwkoop and Faber developmental table (Nieuwkoop and Faber, 1967). Tadpoles were anesthetized with a solution of 200 mg/mL of MS-222 (Sigma) and subsequently decapitated, in agreement with international bioethical recommendations (Close et al., 1996; Ramlochansingh et al., 2014). The Ethics Committee of the University of Concepcion (Concepcion, Chile) approved all experimental procedures carried out during this study, which were performed following the guidelines outlined in the Biosafety and Bioethics Manual of the National Commission of Scientific and Technological Research (CONICYT, Chilean Government). Sense probe negative *in situ* hybridization results are shown in Data Sheet 2. *In situ* hybridizations on paraffin sections were performed as previously described (see Data Sheet 3 and Espinoza et al., 2010; Aldea et al., 2013).

## Results

### Skeletal expression of the major fibrillar collagen genes in *Scyliorhinus canicula* fins and jaws

**Figure 1:**
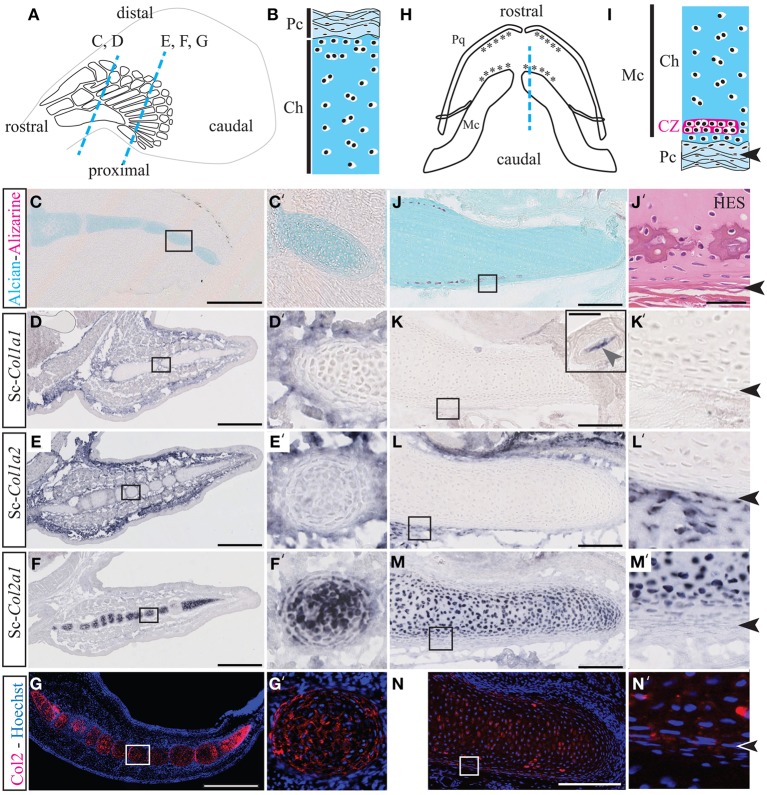
**Cartilage calcification and collagen expression in ***Scyliorhinus canicula*** radials and Meckel's cartilage**. **(A)** Schematic drawing of the pectoral fin anatomy from 7 cm long *S.c.* embryos and of the orientation of the paraffin sections shown in C–G (blue dotted lines). Rostral and caudal refer to the embryonic axis. **(B)** General histology of pectoral skeletal elements, with the center of the cartilaginous element located at the bottom. **(C,C')** Alizarin red and Alcian blue double staining. **(D–F)** Gene expression patterns in the pectoral fin for Sc-*Col1a1* **(D)**, Sc-*Col1a2* **(E)**, and Sc-*Col2a1* **(F)**. **(G)** Immunofluorescence using an anti-Type II collagen (Col2) antibody specifically marks the pectoral fin cartilaginous condensations. **(H)** Schematic drawing of the jaw anatomy from 9 cm-long *S.c.* embryos (ventral view) and of the orientation of the paraffin sections shown in **(J–N)** (blue dotted line). **(I)** General histology of Meckel's cartilage, with the center of the cartilaginous element located at the top. The arrowheads in **(I,J'–N')** demarcate the fibrous perichondrium from the cartilage. **(J)** Alizarin red and Alcian blue double staining. **(J')** Higher magnification of a tesserae located in a similar region as the area boxed in **(J)** and stained with HES. **(K–M)** Gene expression patterns in the jaw for Sc-*Col1a1* [the inset in (**K)** shows a Sc-*Col1a1* positive dermal denticle from the same section], Sc-*Col1a2* **(L)** and Sc-*Col2a1* **(M)**. **(N)** Immunofluorescence using an anti-Type II collagen (Col2) antibody specifically marks the cartilaginous condensations of Meckel's cartilage. Insets in **(C–N)** are shown at higher magnification in **(C'–N')**, respectively. CZ, calcification zone of the tesserae; Ch, chondroctyces; Fb, fibroblasts; Pc, perichondrium; Pq, palatoquadrate. Scale bars: **(C–G)**, 250 μm; **(J–N)**, 100 μm.

The Sc-Col1a1, Sc-Col1a2, and Sc-Col2a1 protein sequences were unambiguously associated to their respective orthology groups by phylogenetic analyses (Data Sheets 4, 5). We examined calcification patterns by Alizarin red, Alcian blue, and HES stainings as well as the expression of Sc-*Col1a1*, Sc-*Col1a2*, and Sc-*Col2a1* in developing *S.c.* fins and jaws (Figure 1).

Alizarin red is specific for high levels of calcium ions and will therefore stain calcified extracellular matrix, while Alcian blue has a strong affinity for glycosaminoglycans of the cartilage matrix. The HES staining classically allows the location of nuclei (dark purple), cytoplasms (pink), and densely organized collagen fibers (orange-pink). Both Safran and the acid aniline dye Eosin will stain the mineralized matrix more intensely than the non-mineralized matrix. Transverse sections through the pectoral fins showed that cartilaginous radials are devoid of calcification both in 7 cm long embryos (Figures 1A–C') and 9 cm long embryos (not shown). By contrast, longitudinal sections of Meckel's cartilage from 9 cm long embryos allowed the detection of tesserae calcification at the cartilage periphery (Figures 1H–J). Tesserae calcification is associated to a darker HES staining of the hyaline matrix surrounding clusters of chondrocytes, and occurs within the cartilaginous scaffold, one or two cell diameters away from the fibrous perichondrium (Figure 1J').

In the pectoral fin, Sc-*Col1a1* and Sc-*Col1a2* are expressed in the fibrous perichondrium and the connective tissue surrounding the cartilaginous elements (Figures 1D–E'), and Sc-*Col2a1* is expressed in the chondrocytes of the cartilaginous matrix of the radials (Figures 1F,F'). In the jaw, we failed to detect Sc-*Col1a1* at the level of Meckel's cartilage, albeit an intense staining was observed in dermal denticles located on the same section and serving as an internal positive control (Figures 1K,K'). Sc-*Col1a2* and Sc-*Col2a1* transcripts were detected, respectively, in the fibrous perichondrium of Meckel's cartilage (Figures 1L,L') and in the chondrocytes of the cartilaginous element (Figures 1M,M'). Immunofluorescence experiments performed on developing *S.c.* fins and jaws further confirmed the cartilage-specific expression of the Sc-Col2a1 protein (Figures 1G,G',N,N'). The punctuated localization of Sc-Col2a1 around the cell body of fin and jaw chondrocytes might result from low levels of expression, and is consistent with the concentration of this protein in the pericellular matrix, as reported in other species (Benjamin and Ralphs, 1991; Mizoguchi et al., 1997; Nah et al., 2001). Taken together, our results support the idea that *S.c.* tesserae growth and calcification occur within a Type I-negative and Type II-positive collagenous microenvironment (Figures 1J–N').

### Skeletal expression of the major fibrillar collagen genes in *Scyliorhinus canicula* vertebrae

**Figure 2:**
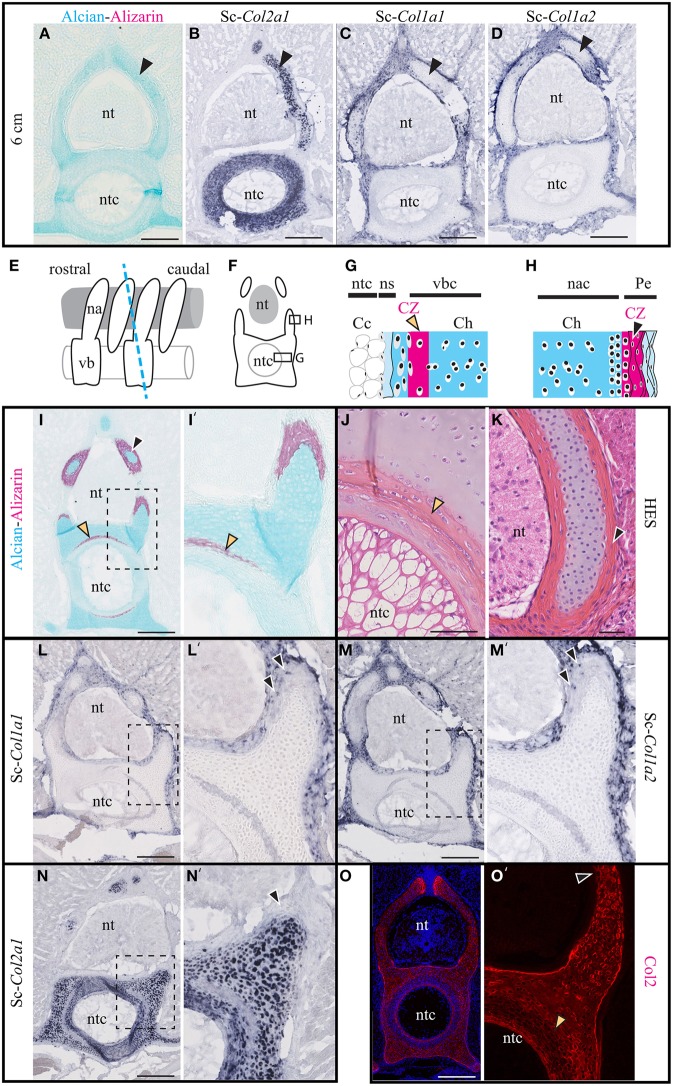
**Cartilage calcification and collagen expression in ***Scyliorhinus canicula*** vertebrae**. **(A–D)** Transverse sections of the vertebrae of 6 cm-long embryos (black arrowheads show the hyaline cartilage of the neural arches). **(A)** Alcian blue and Alizarin red double staining revealing the distribution of the hyaline cartilage and the absence of detectable calcification. **(B–D)** *In situ* hybridizations showing the expression of Sc-*Col2a1*, Sc-*Col1a1*, and Sc-*Col1a2*, as indicated. **(E)** Schematic drawing of the vertebral anatomy from 9 cm-long *S.c.* embryos (lateral view) and of the orientation of the transverse sections (blue dotted line) represented in **(F)** and shown in **(I–O')**. **(G)** General histology of the centrum. **(H)** General histology of the neural arches. **(I)** Alizarin red and Alcian blue double staining. **(J,K)** HES staining of the centrum and of the neural arch. **(L–N)** *In situ* hybridizations showing the expression of Sc-*Col2a1*, Sc-*Col1a1*, and Sc-*Col1a2*, as indicated. Arrowheads in **(L',M')** indicate scattered Sc-*Col1a1* and Sc-*Col1a2* positive cells embedded in the calcified layer of the neural arches. **(O)** Immunofluorescence using an anti-Type II collagen (Col2) specific antibody. Higher magnifications of **(I,L–O)** are shown in **(I',L'–O')** respectively. Orange and black arrowheads show the calcifying matrix of the centrum and neural arches, respectively. Cc, chordocytes; Ch, chondroctyces; na, neural arch; nac, neural arch cartilage; ns, notochord sheath; nt, neural tube; ntc, notochord core; Pe, perichondrium; vb, vertebral body; vbc, vertebral body cartilage. Insets in **(L–O)** are shown at higher magnification in **(L'–O')**, respectively. Scale bars: **(A–D)** 250 μm; **(I,L–O)** 200 μm; **(J,K)** 50 μm.

The transverse sections of 6 cm embryos shown in Figures 2A–D reveal that the *S.c.* vertebrae are cartilaginous, devoid of calcification, and express Sc-*Col2a1* (in chondrocytes of the centrum and the neural arches) and Sc-*Col1a1* and Sc-*Col1a2* (in the perichondrium surrounding all vertebral elements). In the vertebral column of 7 cm-long embryos, Alcian blue stains the cartilaginous vertebrate body and the neural arches (Figures 2E–I'). Alizarin red specifically stains the fibrous perichondrium of the neural arches as well as an internal calcification ring located within the centrum and surrounding the notochord, as reported in other chondrichthyan species (see Figures 2E–K and Peignoux-Deville et al., 1982; Eames et al., 2007). Histologically, the calcified ring of the vertebral body exhibits darker HES staining of the matrix surrounding large cells of chondrocytic appearance (Figures 2G,J). By contrast, cells located in the calcifying extracellular matrix of the neural arches are thin with reduced amount of cytoplasm (Figures 2H,K).

The expression of Sc-*Col1a1* and Sc-*Col1a2* was evident in the fibrous perichondrium and the connective tissue surrounding all vertebral elements (Figures 2L–M') as well as in scattered cells embedded in the calcified layer of the neural arches (arrowheads in Figures 2L',M'). Nor Sc-*Col1a1* neither Sc-*Col1a2* were detected in the calcified layer of the vertebral body (the lighter ring-shaped signal in Figures 2L–M' is identical to the background observed in negative controls, see Data Sheet 2). While Sc-*Col2a1* is expressed in most vertebral chondrocytes, it is significantly downregulated in cells embedded within the calcifying layer of the vertebral body (Figures 2N,N'). Likewise, an anti-type II collagen antibody intensely stained the cartilaginous, non-calcified, vertebral cartilage of the neural arches, and the centrum, as well as a thin layer surrounding the notochord (Figures 2O,O'). In agreement with the *in situ* hybridization results, the calcifying regions of the neural arches and of the vertebral body displayed a much fainter reaction to the Type II collagen antibody (arrowheads in Figures 2O,O'). Taken together, these observations reveal a negative correlation between Sc-*Col2a1* expression and extracellular matrix calcification. By contrast, Sc-*Col1a1* and Sc-*Col1a2* are expressed in all perichondral cells of the vertebrae, regardless of their calcification degree.

### Skeletal expression of the major fibrillar collagen genes in the *Xenopus tropicalis* limb

**Figure 3:**
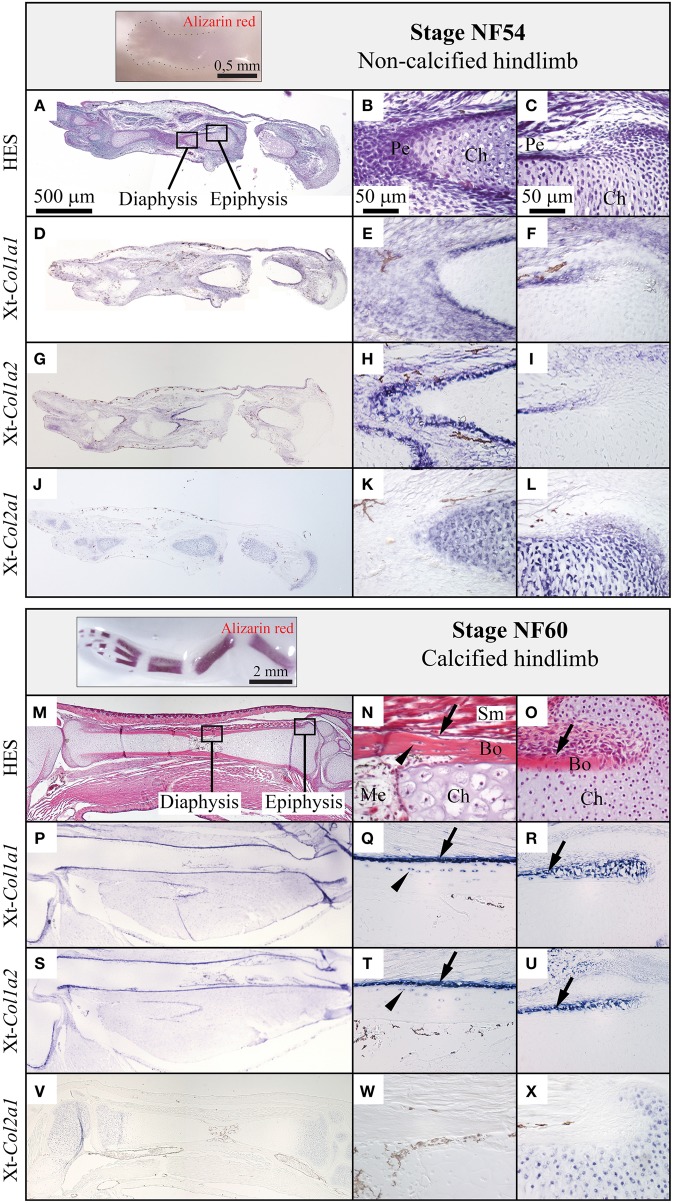
**Comparison of the ***Col1a1, Col1a2***, and ***Col2a1*** expression patterns during ***Xenopustropicalis*** hindlimb development**. Stage NF54 (top panel) or NF60 (bottom panel) hindlimbs were examined by whole mount Alizarin red staining (insets), sectioned along the proximo-distal axis and stained with HES, **(A–C, M–O)** or processed by *in situ* hybridization for the Xt-*Col1a1*, Xt-*Col1a2*, and Xt-*Col2a1* probes, **(D–L, P–X)**. Results are shown for the whole skeletal element (left column, scale bar: 500 μm) and higher magnifications of the diaphysis (middle column, scale bar: 50 μm) and epiphysis (right column, scale bar: 50 μm). Arrows and arrowheads show osteoblasts and osteocytes, respectively. *In situ* hybridization signal is light to dark blue, and brown endogenous *X.t.* pigment cells are visible on most sections. Legend: Bo, bone; Ch, chondrocytes; Me, medulla; Pe, perichondrium; Sm, striated muscles.

We examined the expression of Xt-*Col1a1*, Xt-*Col1a2*, and Xt-*Col2a1* in the diaphysis and epiphysis of *X.t.* hindlimbs both before (stage NF54, Figures 3A–C) and after (stage NF60, Figures 3M–O) ossification. At stage NF54, Xt-*Col1a1*, and Xt-*Col1a2* are most strongly expressed in perichondral cells of developing long bones (Figures 3D–I). At stage NF60, Xt-*Col1a1*, and Xt-*Col1a2* transcripts are robustly detected in osteoblasts and in some osteocytes, albeit more weakly (Figures 3P–U). Finally, Xt-*Col2a1* is expressed in all chondrocytes of NF54 non-calcified cartilaginous elements (Figures 3J–L), and is restricted to the epiphyseal chondrocytes at stage NF60 (Figures 3V–X).

### Histology of the developing *Xenopus tropicalis* vertebrae

**Figure 4:**
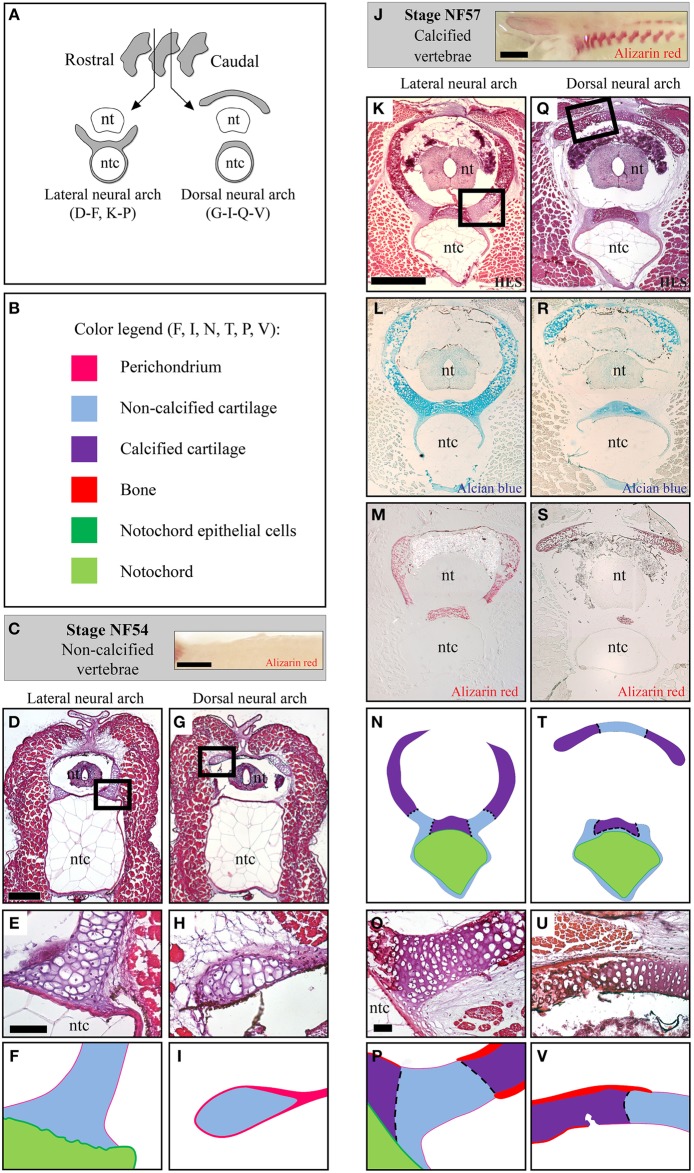
**Histology of the developing ***Xenopus tropicalis*** vertebrae**. **(A)** Schematic drawing of transverse sections running through the lateral or the dorsal region of the neural arches. **(B)** Color code used to represent the distinct skeletal tissues of the *X.t.* vertebrae in **(F,I,N,T,P,V)**. **(C)** Whole mount Alizarin red staining of stage NF54 vertebral column (lateral view, anterior to the left). **(D–I)** Histology of the stage NF54 vertebrae examined with HES **(D,E,G,H)**. **(J)** Whole mount Alizarin red staining of stage NF57 vertebral columns (lateral view, anterior to the left). **(K–V)** Histology of the stage NF57 vertebrae examined with HES **(K,O,Q,U)**, Alcian blue **(L,R)** and Alizarin red **(M,S)**. Insets in **D, G, K** and **Q** are shown in **F, I, P** and **V**, respectively. Panels **E, H, K, O, Q** and **U** are schematized in **F, I, N, P, T** and **V**, respectively. Abbreviations: nt, neural tube; ntc, notochord. Scale bars: 1 mm in **(C,J)**; 250 μm in **(D,G)**; 50 μm in **(E,F)** and **(H,I)**; 500 μm in **(K–N)** and **(Q–T)**; and 50 μm in **(O,P,U,V)**.

Because of the complex shape of the *X.t.* vertebrae, transverse sections either run through the lateral (Figures 4A,D–F,K–P) or the dorsal (Figures 4A,G–I,Q–V) region of the non-calcified (stage NF54, see Figures 4B–I) and calcified (stage NF57, see Figures 4J–V) neural arches protecting the neural tube. At stage NF57, the cartilage matrix is abundant (Figures 4L,R) and displays a pronounced HES and Alizarin red staining co-localizing at the level of the dorsal region underlying the notochord, and within the lateral and dorsal neural arches (see Figures 4K,M,N,Q,S,T). In addition, cartilage calcification and periosteal bone develop in contact to each other (Figures 4O,P,U,V).

### Skeletal expression of the major fibrillar collagen genes in the *Xenopus tropicalis* vertebrae

**Figure 5:**
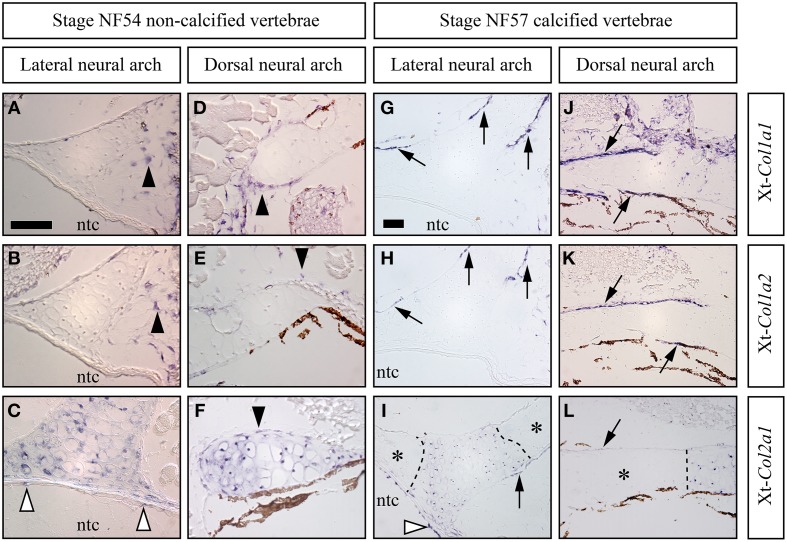
**Skeletal expression patterns the Xt-***Col1a1***, Xt-***Col1a2***, and Xt-***Col2a1*** during ***Xenopus tropicalis*** vertebrae development**. Transverse sections of stage NF54 **(A–F)** and NF57 **(G–L)** vertebrae processed for *in situ* hybridizations using the Xt-*Col1a1*, Xt-*Col1a2*, and Xt-*Col2a1* probes, as indicated. Black arrowheads show loose **(A,B)** or perichondral **(D–F)** cells. White arrowheads in **(C,I)** show *Xt-Col2a1* positive epithelial non-vacuolated cells of the notochord. Arrows point at osteoblasts expressing Xt-*Col1a1* **(G,J)**, Xt-*Col1a2* **(H,K)**, or Xt-*Col2a1* **(I,L)**. In **(I,L)**, calcified, Alizarin red-positive cartilaginous regions are marked by an asterisk and the dotted lines demarcates expression boundaries between *Xt-Col2a1* positive and *Xt-Col2a1*negative chondrocytes. *In situ* hybridization signal is light to dark blue. Brown endogenous *X.t.* pigment cells are also visible in the vicinity of the dorsal neural arch **(D–F, J–L)**. Scale bar in **(A)** represents 50 μm in **(A–F)**; scale bar in **(G)** represents 50 μm in **(G–L)**.

Xt-*Col1a1*, Xt-*Col1a2*, and Xt-*Col2a1* expression patterns were examined in the lateral and dorsal neural arch regions of the vertebrae (see Figures 4E,F,H,I,O,P,U,V). At stage NF54, Xt-*Col1a1*, and Xt-*Col1a2* are expressed in scattered cells of mesenchymal appearance located in the vicinity of the cartilage (Figures 5A,B), as well as in a thin layer of perichondrium surrounding the dorsal neural arch (Figures 5D,E). At this early stage, Xt-*Col2a1* is expressed in all chondrocytes and is also evident in the perichondrium of the dorsal neural arch (Figures 5C,F). At stage NF57, Xt-*Col1a1*, and Xt-*Col1a2* are robustly expressed in osteoblasts lying onto the calcified bone matrix of the vertebrae (arrows in Figures 5G,H,J,K). These osteoblasts also express Xt-*Col2a1*, albeit more weakly than hypertrophic chondrocytes (Figures 5I,L). In chondrocytes, Xt-*Col2a1* is excluded from the Alizarin red-positive regions (asterisk in Figures 5I,L), forming sharp expression boundaries between calcified and non-calcified cartilage (dotted line in Figures 4P,V, 5I,L). In addition, at stages NF54 and NF57, we detected a strong Xt-*Col2a1* staining in the epithelial non-vacuolated cells of the notochord (arrowheads in Figures 5C,I), a known site of *Col2a1* expression in cyclostomes and teleosts (Ota and Kuratani, 2010; Yamamoto et al., 2010).

## Discussion

### Conserved early molecular patterning of the hyaline cartilage and non-calcified perichondrium

In non-calcified *S.c.* skeletal elements, the expression patterns of the *Col1a1*/*Col1a2* (perichondrium) and *Col2a1* (cartilage) genes do not overlap. By contrast, in actinopterygians, *Col2a1* orthologs are expressed in the perichondrium, albeit at lower levels than in cartilage (Albertson et al., 2010; Eames et al., 2012). Likewise, our results in *X.t.* reveal a faint Xt-*Col2a1* expression in the non-calcified perichondrium of the dorsal neural arch at stage NF54. It is likely that more sensitive techniques will help assess the expression levels of Xt-*Col2a1* in the perichondrium of the *X.t.* lateral neural arch or hindlimb, two sites where it was not detected by *in situ* hybridization. Interestingly, Clade A fibrillar collagen members from lamprey and hagfish are expressed both in perichondral cells and in chondrocytes, while the amphioxus ortholog is expressed in chondrocytes and in the mesenchyme located at the tip of regenerating cirri (Zhang and Cohn, 2006, 2008; Zhang et al., 2006; Ota and Kuratani, 2010; Cattell et al., 2011; Kaneto and Wada, 2011). Altogether, these data suggest that the largely complementary expression patterns of *Col1a1*/*Col1a2* (exclusively in the fibrous perichondrium) and *Col2a1* (preferentially in the hyaline cartilage) represent a synapomorphy of non-calcified skeletal elements in jawed vertebrates. It is therefore tempting to propose that the Clade A precursor was expressed in chondrocytes and perichondral cells, and that the functional partitioning of ancestral enhancers was involved in this expression divergence (Force et al., 1999; Zhang and Cohn, 2008). According to this scenario, after the genomic duplications that gave rise to the complete set of Clade A members, the *Col1a1* and *Col1a2* genes would have rapidly lost their cartilage-specific enhancers, while the activity of perichondral *Col2a1* enhancers would have been dramatically reduced, or abolished, in distinct jawed vertebrate lineages.

### *Col2a1* osteoblastic expression was significantly reduced in the tetrapod lineage

**Figure 6:**
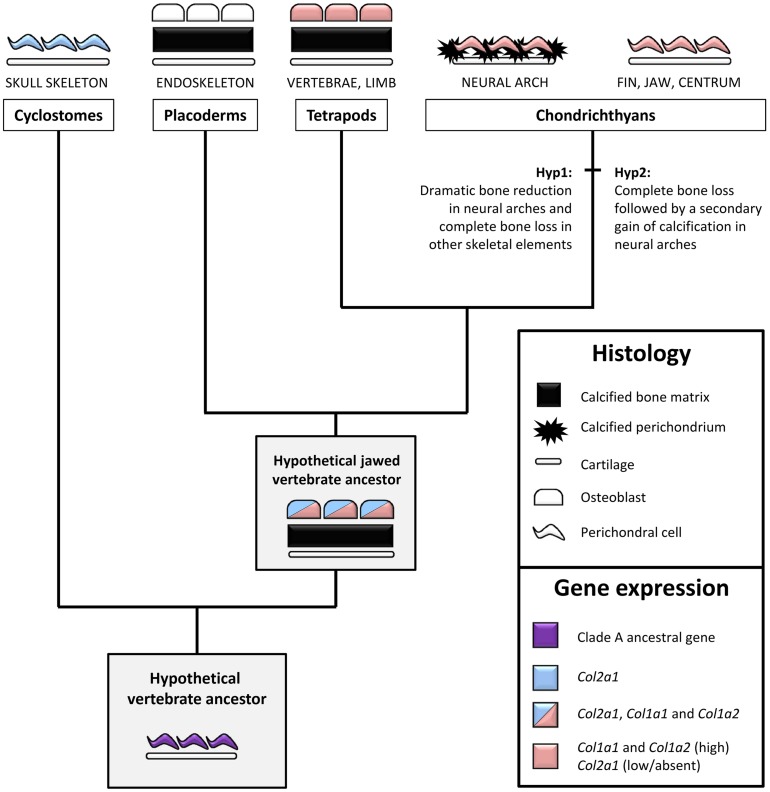
**An evolutionary scenario for bone formation and perichondral calcification in jawed vertebrates**. Bone/perichondrium histology and gene expression patterns were mapped onto a simplified vertebrate phylogenetic tree to deduce ancestral states and polarize evolutionary change. We propose that the ancestral Clade A fibrillar collagen gene (i.e., before the duplications that produced the distinct member of this family) was expressed in the non-calcified perichondrium. This expression pattern was inherited by the unique cyclostome fibrillar collagen gene which is more closely related to the *Col2a1* subgroup. In jawed vertebrates, perichondral cells and osteoblasts maintained high levels of *Col1a1* and *Col1a2* while the *Col2a1* osteoblastic expression was dramatically reduced in most (but not all) lineages. The presence of bone in placoderms and tetrapods supports the idea that the calcified fibrous perichondrium observed in some chondrichthyan species either represents bone evolutionary remnants (Hypothesis 1) or a secondary gain of calcification (Hypothesis 2). Osteocytes have been omitted for the sake of simplicity. See text for details.

We detected *X.t. Col2a1* transcripts in osteoblasts of the vertebrae, albeit they displayed a weaker *in situ* hybridization signal than hypertrophic chondrocytes present on the same section (Figures 5I,L), which is consistent with expression results obtained with primary cultures of *X.t.* osteoblasts (Bertin et al., 2015). While *Col2a1* is traditionally considered to be a chondrocyte-specific marker (Kobayashi and Kronenberg, 2005; Hartmann, 2009), its robust osteoblastic expression has been reported in embryos from several species of actinopterygian fishes (Benjamin and Ralphs, 1991; Albertson et al., 2010; Eames et al., 2012). The moderate *Col2a1* expression levels described in the clawed frog (this study), chick (Abzhanov et al., 2007) and mouse (Hilton et al., 2007) therefore support the idea that the osteogenic transcription of *Col2a1* was significantly reduced in the tetrapod lineage, and almost completely abolished in mammals (Figure 6).

### *Scyliorhinus canicula* neural arches, tesserae, and centrum calcification occur in distinct molecular contexts

Our results reveal that at least three skeletal sites expressing different combinations of collagen genes are associated with robust *S.c.* calcification in: (i) the fibrous perichondrium of the neural arches, (ii) the tesserae developing in Meckel's cartilage, and (iii) the compact cartilage embedded within the vertebral bodies.

In neural arches, the cartilaginous scaffold is surrounded by a fibrous perichondrium whose matrix is highly calcified and devoid of Col2 protein, and whose cells express Sc-*Col1a1* and Sc-*Col1a2* and no detectable levels of Sc-*Col2a1* (Figures 2, 5). The evolutionary relationship between this calcified perichondrium and the osteichthyan bone has remained enigmatic and controversial (Peignoux-Deville et al., 1982; Eames et al., 2007; Zhang et al., 2009; Ryll et al., 2014). In the light of fossil evidence demonstrating that extant chondrichthyans are quite derived, having lost the perichondral bone surrounding the cartilaginous elements (Coates et al., 1998; Donoghue and Sansom, 2002), two hypotheses might account for the unusual calcification pattern observed in neural arches (Figure 6). On the one hand, it is possible that the perichondral bone was dramatically reduced to some evolutionary remnants of calcified fibrous perichondrium located in the neural arches (hypothesis 1). In this case, the cells involved in matrix calcification would correspond to highly derived osteoblasts having lost many crucial cellular features typically observed in osteichthyans, such as the ability to organize as a polarized pseudoepithelium (Izu et al., 2011; Liu et al., 2011). On the other hand, the perichondral bone might have been completely lost, and secondarily compensated by an independent ability to calcify the perichondral extracellular matrix (hypothesis 2). Below, we discuss two complementary strategies that might help resolve this issue. Firstly, a broader phylogenetic sampling is required to precisely assess the occurrence of a calcified perichondrium in neural arches, which currently seems to be limited to some chondrichthyan species. For instance, the skeleton of holocephalans displays little or no calcified tissue (a ring-shaped calcification of the centrum is reported in some fossil holocephalan and in the extant genus *Chimaera*) while batoids (rays and skates) have a tesserae-based calcification at the surface of their vertebral units (Reynolds, 1897; Goodrich, 1930; Zangerl, 1981). Secondly, it will be important to investigate the nature of the *Col1a1* and *Col1a2* positive cells embedded within the mineralized matrix (Figures 2E',F'). Indeed, such cells have been proposed to be osteocytes (Peignoux-Deville et al., 1982), which is consistent with the fact that cellular bone evolved before the origin of the jawed vertebrates (Donoghue and Sansom, 2002; Donoghue et al., 2006; Sanchez et al., 2013). Extensive phenotypical and molecular similarities between the scattered cells embedded within the *S.c.* calcified perichondrium and osteichthyan osteocytes would support their homology, and, therefore, the aforementioned hypothesis 1.

**Figure 7:**
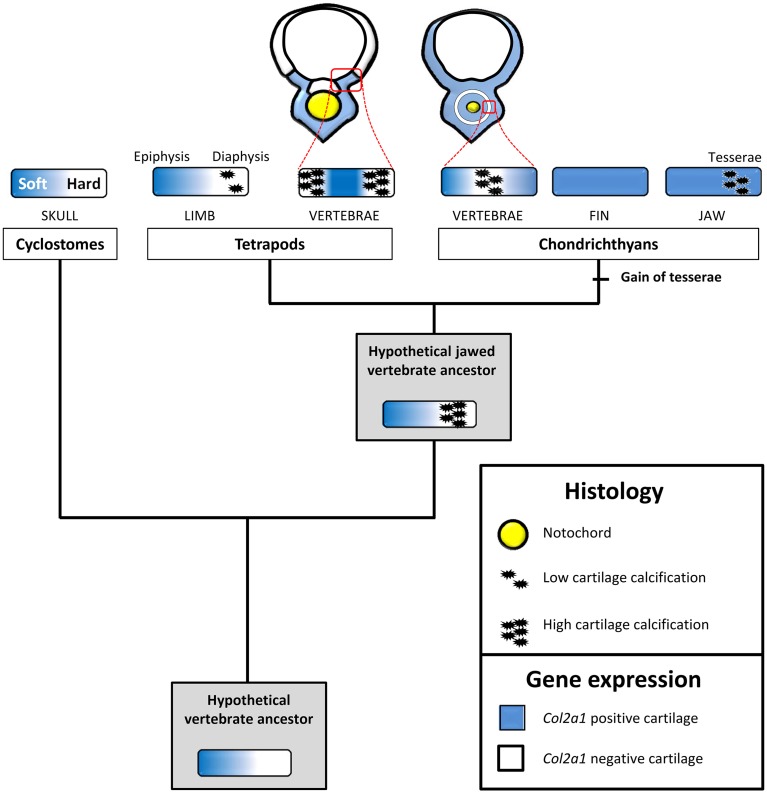
**An evolutionary scenario for cartilage calcification in jawed vertebrates**. Expression patterns and cartilage matrix calcification were mapped onto a simplified vertebrate phylogenetic tree to deduce ancestral states and polarize evolutionary change. We propose that, in the last vertebrate common ancestor, the expression of *Col2a1* experienced a strong downregulation in maturing, non-calcified, cartilaginous regions. This downregulation was subsequently inherited by distinct vertebrate lineages, and is associated to hard cartilage in cyclostomes and to calcified cartilage in jawed vertebrates. The chondrichthyan and osteichthyan representatives analyzed in this study display a calcified *Col2a1*-negative vertebral cartilage, a likely jawed vertebrate synapomorphy. Tesserae calcification, a recent chondrichthyan innovation, occurs in the absence of *Col2a1* downregulation. Perichondrium and bone have been omitted for the sake of simplicity. See text for details.

Another site of calcification in *S.c.* corresponds to the developing tesserae embedded in Meckel's cartilage, a process classically described to occur at the surface of the cartilaginous skeletal piece (Kemp and Westrin, 1979; Dean et al., 2009). As we show here, the onset of this type of calcification takes place in a Col2-positive context, within the cartilaginous scaffold (Figures 1, 6). We failed to detect *Col1a1*/*Col1a2* expression in the chondrocytes neighboring the mineralized matrix, suggesting that the cellular processes involved in matrix calcification are very different from what has been described in osteichthyan bone or chondroid bone (Mizoguchi et al., 1997). This type of calcification is well developed in extant batoid and selachimorph species, and is also known in fossil holocephalan species (Grogan and Lund, 2000; Finarelli and Coates, 2014) and, therefore, is considered to be an early evolutionary innovation of the chondrichthyan lineage (Figure 7).

Below, we will discuss the third type of calcification mechanism, which occurs in the *Col1a1*/*Col1a2* negative *S.c.* vertebral cartilage experiencing a drastic *Col2a1* downregulation, in the light of the striking similarities that it shares with the *X.t.* vertebrae.

### An ancient type of calcified vertebral cartilage associated to the down-regulation of *Col2a1*

The tetrapod hyaline cartilage calcifies its extracellular matrix, albeit to a much lesser extent than the bone tissue (Claassen et al., 1996; Khanarian et al., 2014) and, therefore, only weakly stains with Alizarin red (Kirsch et al., 1997). Here, we report an unusual type of calcified cartilage displaying remarkable similarities between *X.t.* and *S.c.* at three distinct biological levels: (i) anatomically, this cartilage is located in the vertebrae of both species, and, at least at the stages analyzed, in no other skeletal elements; (ii) from an histological perspective its robust calcification is reflected by intense Alizarin red and HES stainings; (iii) molecularly, both types of cartilages are *Col1a1*/*Col1a2* negative and probably experience a *Col2a1* downregulation, because in both species all cells of the vertebral cartilage express *Col2a1* during early, non-calcified, developmental stages (see Figures 2B,N,N', 5C,F,I,L). In this respect, both types of vertebral cartilages seem to recapitulate the initial phase of endochondral bone formation typically seen in tetrapod long bones, during which proliferative chondrocytes progressively downregulate the expression of *Col2a1*, undergo hypertrophy, and calcify their extracellular matrix (Figure 7). Our observations, combined to data from mouse (Chandraraj and Briggs, 1988), and lizards (Lozito and Tuan, 2015), suggest that an calcified form of vertebral cartilage was present in the last common ancestor of jawed vertebrates, at least as a transitory developmental process.

As vertebral developmental processes are highly variable, homology relationships between the calcified ring surrounding the *S.c.* notochord and the calcified cartilage of the *X.t.* vertebrae cannot be inferred (Fleming et al., 2015). Rather, we propose that the genetic programme involving a downregulation of the *Col2a1* gene predates the emergence of the last vertebrate common ancestor, and was subsequently co-opted and modified to produce a variety of novel non-calcified (Zhang and Cohn, 2006; Zhang et al., 2009) and calcified (Hogg, 1982; Claassen et al., 1996; Janvier and Arsenault, 2002; Porter et al., 2007) cartilaginous structures (Figure 7). One intriguing possibility is that the ancient, *Col2a1*-negative, calcified cartilage present in the last common ancestor of jawed vertebrates later came to play a key role in the subsequent elimination of cartilaginous matrix and its replacement by bone tissue. In this respect, it might have served as a crucial pre-patterning step contributing to the emergence of endochondral ossification commonly observed in tetrapods and whose precise origin still remains to be determined. In the future, a comprehensive comparison of gene expression signatures between cell types present in diverse skeletal tissues, anatomical locations, developmental stages, and species will provide a solid basis to unravel the complex and fascinating evolutionary history of the vertebrate skeleton.

### Conflict of interest statement

The authors declare that the research was conducted in the absence of any commercial or financial relationships that could be construed as a potential conflict of interest.

## Supplementary material

Supplementary Table 1 **list of *Scyliorhinus canicula* and *Xenopus tropicalis* specific primers**.

Data Sheet 1 **Sequences of the *S.c.* clones**.

Data Sheet 2 **Sense probe negative *in situ* hybridization results for *Scyliorhinus canicula* and* Xenopus tropicalis***.

Data Sheet 3 ***X.t. in situ* hybridization protocol**.

Data Sheet 4 **Molecular Phylogenetic analysis of gnathostome Clade A fibrillar collagen genes**. Phylogenetic relationships were inferred and the tree with the highest log likelihood (-7413.8950) is shown. The percentages of trees in which the associated taxa clustered together are indicated. The tree is drawn to scale, and branch lengths correspond to the number of substitutions per site. Orthology groups (OG) are identified as blue circles, and *S.c.* sequences are shown in red. The tree was rooted according to (Zhang and Cohn, 2008).

Data Sheet 5 **Fibrillar collagen sequences**.
