# Supplementary material for: Molecular footprinting of skeletal tissues in the catshark Scyliorhinus canicula and the clawed frog Xenopus tropicalis identifies conserved and derived features of vertebrate calcification
Source: Front Genet. 2015 Sep 15;6:283. doi: 10.3389/fgene.2015.00283 (PMC4584932; doi:10.3389/fgene.2015.00283)
Supplement: Supplementary Table 1 — list of Scyliorhinus canicula and Xenopus tropicalis specific primers. [file Table1.DOCX]

**Supplementary table 1:** list of *Scyliorhinus canicula* and *Xenopus tropicalis* specific primers.

| ***Scyliorhinus canicula* primers** | | | |
| --- | --- | --- | --- |
| **Name** | **Sequence (5’-3´)** | **Size (bp)** | **NCBI Accession number** |
| Sc-Col1a1-F  M13-F (end of clone) | CTGCAGGTGACATTTTGCCA  ACACAGGAAACAGCTATGAC | 748 | **KT261785** (Probe region overlaps with EU241868.1) |
| Sc-Col1a2-F  Sc-Col1a2-R | ATCCTGCTCGCTCATGTCGT  TGAAACAGACTGGGCCAATG | 630 | **KT261784** (Probe region overlaps with EU241869.1) |
| Sc-Col2a1-F  Sc-Col2a1-R | GCTGGTCTATCACAACCTGA  ACCTGTATTCGATGACTGTC | 691 | Probe region included within **EU241867.1** |
| ***Xenopus tropicalis* primers** | | | |
| **Name** | **Sequence (5’-3´)** | **Size (bp)** | **NCBI Accession number** |
| Xt-Col1a1-F  Xt-Col1a1-R | GGCACCCATGGATATCGGAG  CGCTGTCAACTTTTGGGTCG | 1197 | **NM_001011005.1** |
| Xt-Col1a2-F  Xt-Col1a2-R | GAGGGCAACAGCAGATTCAC  ACGGAAAAGTGAGTCGTAAGC | 669 | **NM_001079250.1** |
| Xt-Col2a1-F  Xt-Col2a1-R | GGCTGCAAGAAACACACTGG  CTCCCAGATGCAGAACCCAG | 901 | **NM_203889** |
