# Supplementary material for: Molecular footprinting of skeletal tissues in the catshark Scyliorhinus canicula and the clawed frog Xenopus tropicalis identifies conserved and derived features of vertebrate calcification
Source: Front Genet. 2015 Sep 15;6:283. doi: 10.3389/fgene.2015.00283 (PMC4584932; doi:10.3389/fgene.2015.00283)
Supplement: Data Sheet 1 — Sequences of the S.c. clones. [file DataSheet1.DOCX]

Sc-*Col1a1* probe

CTGCAGGTGACATTTTGCCATATACCACTGCAGAGAAAAACTGAAAGATTGTATGTACCTTTTTCAGGTGGCAAAATAAATTTGAAAAAATGTCAAAATCTTGTTATTGTCCTTCTACATGTGGTGAGAAAGCATCGTTTCTTTGCTATTTTGTAGTGCAGAAGAGTAAAATCCTCACATCTTCCAATACAAAACTTATAGGGCTACCTCACACAAAATGTCACCTTCTGTTTTAGCAAGCCACAGTGAGTCAAAAAGCAGTGCTGGGATAGGTTATACAGCTATAAGAAAAATATATAAAAAGAAAGGTGCTATTTATGCTTAGGTTCTTAAAGCTGTGCAGCGCAGCACAGAAAGCTTGAAGAACCTCTGACTGGTGCTAAAGAAACATTCCTCCCTGCCTAGGAATACGGTGAATTTCTTTTCCAAATGAGGTGCTATTTAGAAGTGTCTGTCTGTACACAAGGAGAAAACTTTTATGTGCAACAAAACATCGCAAACTACCTGTCTCTTTCAAAGTTAAGCATGTGGATCCCTATCTCCCTTTGCCATTCCTGTTTCATCATCTCAATGCCCTTTTTGTGCTTCTTTTTGGCAATACCCCTTTTTTTCTACTTTAGAGTGGAAAAATAGAGGCAGACATTTCTGGGGCAAGTACATGGATATGTACCTATTTTGTATATGTATAATAATTTGAGATGTTTTTAATTATTTTGAATGCTGAAATAAAGCATGTTTAAATG

Sc-*Col1a2* probe

ATCCTGCTCGCTCATGTCGTGACCTGAGACTCAGCCATCCAGAATGGAAGTCCGGTTACTATTGGATCGATCCCAATCAAGGATGTATCATGGATGCCATCAGAGTTTATTGTGACTTTGTATCAGGCGAGACTTGTGTACATGCAAACCCAGACATCATAGAACGCAGGAACTGGTGGAATAACAAGACTCCCAAAGAAAAGAAACACGTATGGTTTGGTGAAACTATCAATGATGGTGCCCAATTCACTTACAACGATGAACACATTAGTCCATTTATTATGGGTACCCAGCTGACCTTCCTGCACCTTCTCTCCAATGAAGCCGCACAGAATATCACCTATCATTGTAAGAACAGTGTAGCATATATGGATGAGGAGACTGGAAACCTGAAAAAGTCAGTGATCCTACTTGGTTCCAATGATGTTGAACTCCGAGCTGAAGGTAACAGCAGATTCACTTACAGCGTTCTGGAAGACGGATGCACAAGACATACTGGTGAATGGAGTAAGACAGTCATTGAATACAGGACACAGAAAACATCTCGCTTGCCATTTATGGACATTGCACCTTTGGACATTGGTGGCCCTGAACAAGAATTTGGTTTGGACATTGGCCCAGTCTGTTTCA

Sc-*Col2a1* probe

GCTGGTCTATCACAACCTGAGAAAGCTCCTGATCCACTCCGATACTTCCGGGCTGACCAGGCTGCTCCGTTCCTTCGTCAACATGATGCCGAGGTTGATGCCACTCTGAAATCCCTTAACAACCAGATTGAAAATATCCGTAGCCCAGAAGGAAGCAAGAAAAACCCAACCCGTACCTGCCGAGACCTGAAACTTTGTCACTCAGACTGGAAGAGCGGTGATTACTGGATCGATCCTAACCAGGGCTGCACACTCGATGCCATCAAGGTCTTCTGTAACATGGAAACTGGTGAGACCTGTGTCTATCCCAATCCAACAAGCATTCCACGAAAGAACTGGTGGACCAGCAAGGGTAAAGACAAGAAACATGTATGGTTTGGCGAGACTATGAATGGTGGTTTCCATTTCAGCTATGGTGATGGTAGTTTGACAGCCAACACTGCTGCCATCCAGATGACATTCCTGCGTCTGCTGTCCACTGAAGCCTCCCAAAACATCACCTATCACTGCAAAAATAGCATTGCCTACATGGATAGAGCTTCTGGTAACCTGAAGAAGGCCCTTCTATTGCAAGGCTCAAATGACATTGAAATCAGAGCTGAAGGAAACAGCAGGTTCACATACACGGTCTTGGAGGATGGTTGCACGAAACATACTGGCAAGTGGGGCAAGACAGTCATCGAATACAGGT
