# Supplementary figures and images for: Molecular footprinting of skeletal tissues in the catshark Scyliorhinus canicula and the clawed frog Xenopus tropicalis identifies conserved and derived features of vertebrate calcification
Source: Front Genet. 2015 Sep 15;6:283. doi: 10.3389/fgene.2015.00283 (PMC4584932; doi:10.3389/fgene.2015.00283)

*Scyliorhinus canicula*

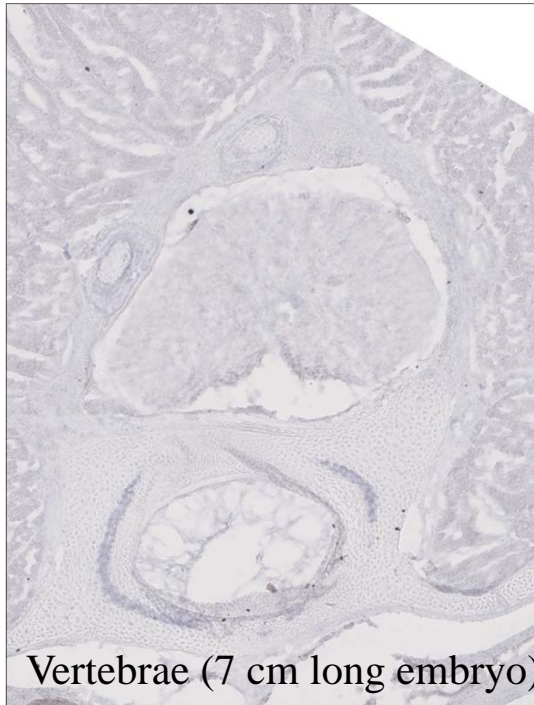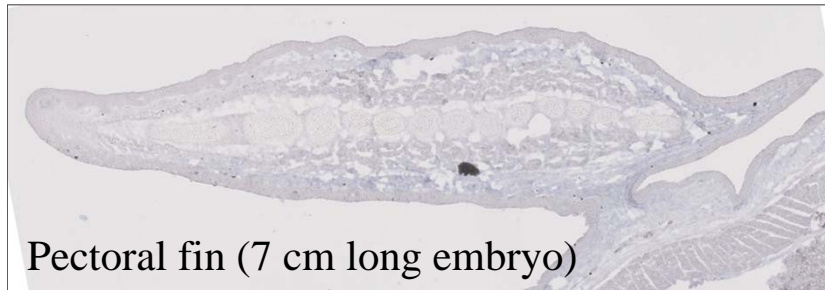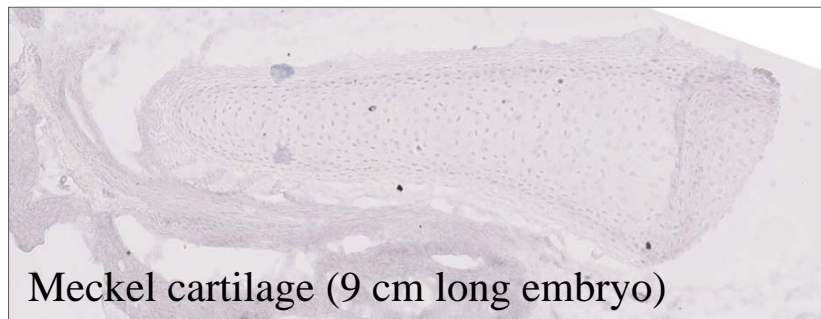

*Xenopus tropicalis*

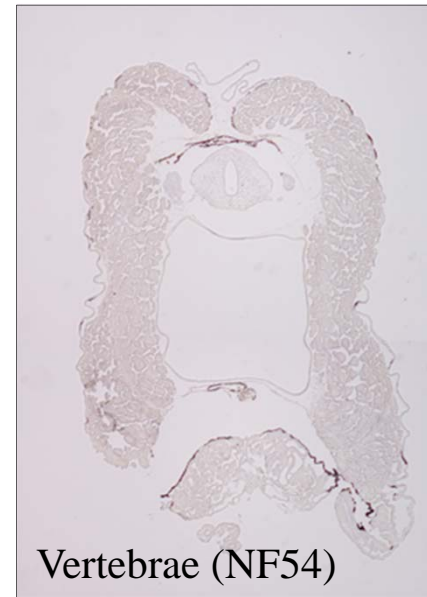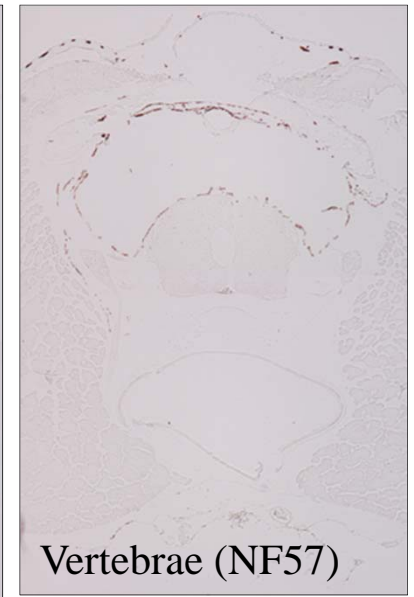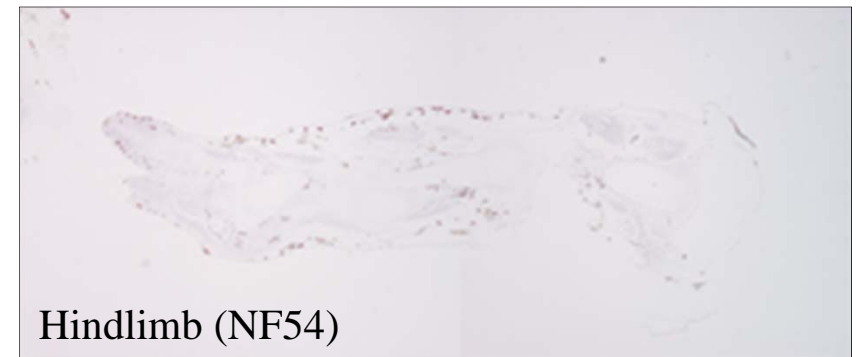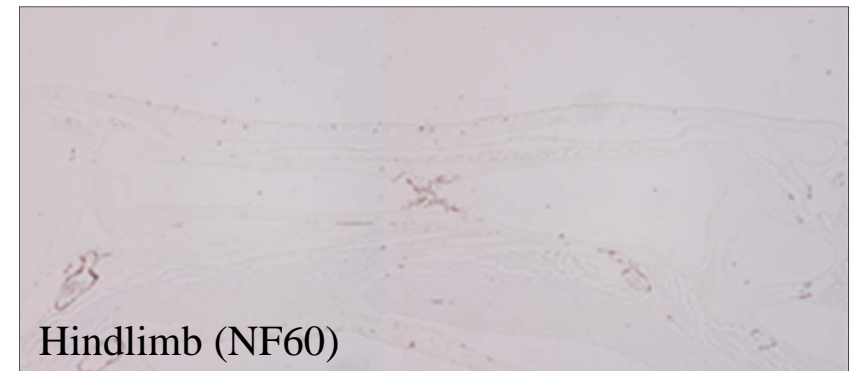

Supplement: Data Sheet 2 — Sense probe negative in situ hybridization results for Scyliorhinus canicula and Xenopus tropicalis. [file DataSheet2.PDF]
