# Supplementary material for: Molecular footprinting of skeletal tissues in the catshark Scyliorhinus canicula and the clawed frog Xenopus tropicalis identifies conserved and derived features of vertebrate calcification
Source: Front Genet. 2015 Sep 15;6:283. doi: 10.3389/fgene.2015.00283 (PMC4584932; doi:10.3389/fgene.2015.00283)
Supplement: Data Sheet 3 — X.t. in situ hybridization protocol. [file DataSheet3.PDF]

in situ hybridization protocol for paraffin sections of *Xenopus tropicalis* tadpole skeletal elements

Getting started: 2
Day 1 4
Day 2 5
Day 3 5
Reagents 6
General Comments - Working with RNA 7
Fixatives 8
How to prepare TEA - Acetic anhydride? 9
Bench table – Day 1 10
Bench table – Day 2 11
Bench table – Day 3 12

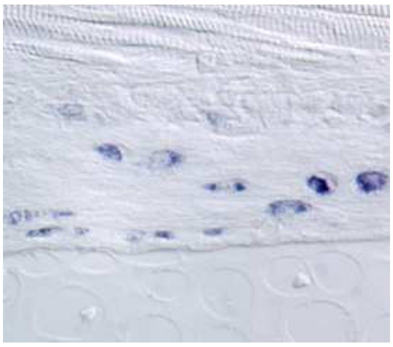

Dentin Matrix Protein1
(Osteocyte-specific)

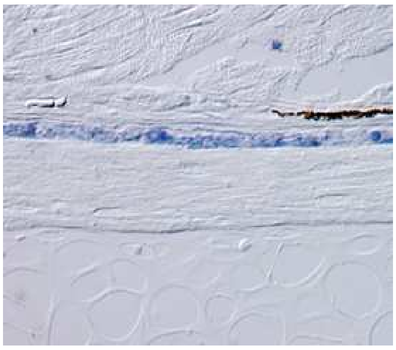

Osteocalcin
(Osteoblast-specific)

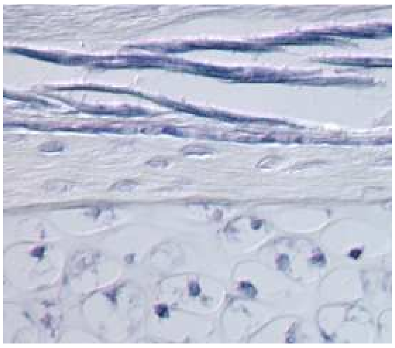

Collagen 8a2
(Osteoblasts, osteocytes, chondrocytes and striated muscle fibers)

Getting started:

<u>Prepare DEPC-treated water:</u>
Add 1 mL of DEPC to 1 liter of nanopure water. Incubate O/N at 37°C and autoclave.

<u>Prepare the following solutions in DEPC-treated water:</u>

PBS 20X:

SALT	MW (g/mol)	Final concentration	g. for 500mL
NaCl	58.44	2.74 M	80.1
KCl	74.55	0.054 M	2
Na_2HPO_4	141.96	0.2 M	12.6
KH_2PO_4	136.09	0.04 M	2
Adjust to pH 7.4 and fill to 500 mL with DEPC-treated water. Autoclave.			

MORSE: 10% sodium citrate; 20% formic acid in DEPC-treated water. *(See Shibata et al. 2000, Assessment of decalcifying protocols for detection of specific RNA by non-radioactive in situ hybridization in calcified tissues. Histochem Cell Biol 113(3): 153-159)*

PFA 4%: See page 8!

MEM 10x: 1M MOPS; 20mM EGTA; 10mM $MgSO_4$

MEMFA: 1 volume of MEM 10x; 1 volume of Formaldehyde 37%; 8 volumes of DEPC-treated water.

TNE buffer (Tris/NaCl/EDTA): 10mM Tris pH7.5; 500mM NaCl; 1mM EDTA.

0.1M TEA and 0.25% acetic acid: See page 9!

HYBRIDIZATION BUFFER:

0.4 g	Roche Blocking reagent
20 mL	Formamide
10 mL	SSC 20 X
**Heat for 1 hour at 65ºC until dissolved, then add:**	
4.6 mL	DEPC-treated water
4 mL	Yeast RNA (10 mg/mL)
80 uL	Heparin (50 mg /mL)
0.4 mL	10 % Tween
0.4 mL	CHAPS 10 %
0.4 mL	EDTA 0.5 M pH 8
**Store at -20º C**	

Sample preparation.

1. Anaesthetize tadpoles following bioethical rules recommended for amphibians *(See Close, Banister, Baumans, Bernoth, Bromage, Bunyan, Erhardt, Flecknell, Gregory, Hackbarth, Morton and Warwick. Recommendations for euthanasia of experimental animals: Part 1, 1996, Laboratory animals, Vol 30, pp 293-316).*
2. Dissect the tissue in 0.6X HBSS (correct osmolarity for *Xenopus tropicalis* live tissues).
3. Fix the samples in PFA/PBS 4% or in MEMFA (O/N) at 4ºC.
4. Remove the fixative; then add 500 µL of MORSE solution and gently shake for 1 to 3 hours at RT (**IMPORTANT:** this step is to demineralize skeletal elements, and should be omitted for soft tissues).
5. Remove the MORSE solution and progressively dehydrate in EtOH as follow: 50% EtOH in DEPC-treated water for 5min; 100% EtOH for 5min; EtOH 100% 20min, and store at -20ºC in EtOH 100%.

Paraffin inclusion.

1. Remove EtOH 100%, then add 500 µL of Xylene (work under the fume hood). Gently shake for 30'.
2. Replace with a 1V/1V solution of Xylene/paraffin and incubate at 60ºC for 15min.
3. Replace with 100% paraffin, incubate 1 hour at 60ºC (repeat 3 times).
4. Place the sample in a paraffin block (cassette). Leave O/N at 4°C.
5. Cut the sample at 7 µM thickness with a microtome. For bone sections it is recommended to change the microtome blade regularly.

Sample mounting:

1. Place a drop of DEPC-treated water on a slide, at 50-55 °C on a heating block.
2. Place the samples on top of the drop, then wait until the sample is well stretched, and gently remove the water.
3. Incubate the slide at 37°C O/N.

Materials preparation:

All materials (slide racks, moist chamber, etc) **must to be treated in NaOH 0,5%** to eliminate RNAses. Immerse the materials in NaOH 0,5% the day before starting your hybridization *in situ*, extensively rinse in DEPC-treated water before use.

Solutions that can be prepared in normal nanopure water (i.e. non-DEPC treated).

Buffer 1: For 300 ml, mix 1M Tris-pH7.5 (30 ml), 3M NaCl (15 ml) and 255 ml of nanopure water.

Alkaline phosphatase buffer (pH 9,5) : 0.1M Tris pH 9.5 (5ml 1M Tris); 0.1M NaCl (1.66ml 3M NaCl); 50mM $MgCl_2$ (2.5ml 1M $MgCl_2$); 0.1% Tween (500µl Tween 10%)and fill to 50ml with nanopure water.

Day 1

We perform the washes and incubations in slide racks of 50 mL.

All the solutions are prepared with DEPC-treated water

1. Incubate for 1 hr at 60°C to remove the excess of paraffin (place the slide in a vertical position).
2. Cool the slide at RT for 5 min.
3. Place the slide in Xylene for 5 min (Perform twice. Use two different Xylene slide racks).
4. Rehydrate as follow:
    - 5min in EtOH 100%
    - 5min in EtOH 95% / PBS
    - 5min in EtOH 75% / PBS
    - 5min in EtOH 50% / PBS
    - 5min in EtOH 25% / PBS
    - 5min in PBS 100% (twice)
5. Post-fix in PFA 4%/PBS for 20 min at RT.
6. Wash the samples with PBS/Tween 0.1% for 5 min (twice).
7. Treat slides with Proteinase K (10ug/mL) / PBS at 37 °C for 30min. (**IMPORTANT:** it is crucial to optimize this step, as weak PK treatments will result in a drastic decrease of signal, and excessive treatments will damage your sample. We recommend to calibrate the concentration and duration of PK treatments)
8. Post-fix with PFA 4%/PBS for 5 minutes at RT (Reuse fixative from step 5).
9. Wash with PBS/Tween 0.1% for 5 min (twice).
10. Acetylate with Trietanolamine (TEA) 0.1M pH 7-8 and 0,25%acetic anhydride for 5 min at RT. (**IMPORTANT**: This treatment removes the charge on the slide and tissue to avoid probe binding. Acetylation of the positively charged amino groups in the proteins of the tissue decreases background binding of the negatively charged RNA probe. It also appears to inactivate RNases and may help in producing a strong signal)
11. Wash in PBS/Tween 0.1% at RT (twice)
12. Rinse in PBS for 10 seconds.
13. Prehybridize with hybridization buffer for 2-3 hr at 60°C.
14. Hybridization: denature the probes (150 ng) in 150 µl hybridization buffer by heating at 80°C for 5 min, then quickly chill on ice for 5 min.
15. Incubate the samples with hybridization mix (probe + hybridization buffer) at 60°C O/N. Place your slides in a moist chamber and cover your samples with a coverslip to prevent evaporation as follow:

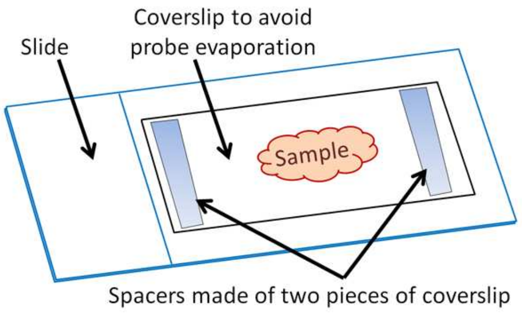

Day 2

All the solutions are prepared in normal nanopure water (*i.e.* non-DEPC treated).

1. Remove the coverslips dipping the slide in SSC 4X-0.1% CHAPS at 60°C.
2. Wash in SSC 2X-0.1% CHAPS/ 50% formamide for 30 min. Place the slide in horizontal position then cover with the solution. Incubate at 60°C. To avoid evaporation, add more solution every 10 min.
3. Wash in TNE buffer for 10 min at 37 °C.
4. Wash in TNE + RNase A (20ug/mL) for 30 min at 37°C.
5. Wash in TNE for 10 min at 37°C.
6. Wash in SSC 2X 0,1% CHAPS for 20 min at 60°C.
7. Wash in SSC 0.2X 0,1% CHAPS for 20 min at 60°C (twice).
8. Wash in 50% (SSC 0.2X-0.1% CHAPS)/50% buffer1 for 5 min at RT.
9. Wash with Buffer 1 for 5 min at RT.
10. Block for 1 hr at RT with Buffer 1 + 20% goat serum.
11. Incubate with antibody (anti-DIG-AP) 1:2000 in Buffer 1 + 20% goat serum O/N at 4°C, in a moist chamber.

Day 3

1. Wash in Buffer 1 + Tween 0,05% in deionized water for 10 min at RT (4 times).
2. Wash in alkaline phosphatase buffer (pH 9,5) for 5 min at RT (twice).
3. Replace with coloration solution (1 tablet of NBT/BCIP dissolved in 10 ml of nanopure water). To avoid sample dying or evaporation, add more coloration solution regularly.
4. When appropriate, stop the reaction by washing for 10 min in PBS at RT (3 times).
5. Fix in MEMFA for 1hr at RT.
6. Dehydrate samples progressively by washing your slides as follow:
   - 5 min in EtOH 100% I
   - 5 min in EtOH 100% II
   - 5 min in Xylol
   - 5 min in Carbol-Xylol
   - 5 min in Xylol II
   - 5 min in Xylol III
   - 5 min in Xylol IV
7. Mount in Entellan.

Reagents

	Supplier	Reference number
Acetic anhydride	Sigma-Aldrich	33214
Anti-Digoxigenin AP conjugate	ROCHE	11093274
CHAPS	Merck-Calbiochem	220201-10GM
DEPC	Sigma-Aldrich	159220-100G
Entellan	Merck	1079610100
Formaldehyde 37%	Sigma-Aldrich	F8775
Formamide	Merck-Calbiochem	344206
Goat serum	USBiological	S1003-45
HBSS	Thermo Scientific Hyclone	SH30031.03
Heparin	Merck-Calbiochem	375095-100KU
Microtome Blades for bone	Sakura	4685
MOPS	Merck-Calbiochem	475922
NBT/BCIP tablet	Roche	11697471001
Paraffin	Merck	111609
Proteinase K	Life Technologies-Ambion	AM2542
RNaseA	USBiologicals	R2011
Roche Blocking Reagent	Roche	11096176001
Slides	Fisher-Scientific	12-550-15
SSC 20 X	Life Technologies-Invitrogen	15557044
Triethanolamine (TEA)	Merck	1083790250
Yeast RNA	Sigma-Aldrich	R6625-25G

General Comments - Working with RNA

RNA is sensitive to degradation by RNases. As well as being found on human skin and produced by a micro-organisms, RNases can be very hard to destroy: They are for instance resistant to temperatures well over 100°C. For these reasons a number of precautions to avoid contamination are usually used.

1. Wear gloves

2. Keep special stocks of tips and tubes for RNA work.

3. Treat solutions with diethylpyrocarbonate (DEPC). Added to 0.1% and left overnight, this destroys RNases. Autoclaving then breaks down DEPC to H_2O and CO_2. Be careful: **TRIS is incompatible with DEPC** so you should always prepare Tris solutions with DEPC treated H20. When you weigh powder don't use spatula, use either disposable scalpel or pour the powder.

The solutions you can treat with DEPC are (you make the solutions with bi-distilled H_20 and then you add 0.1% DEPC, you leave them stirring overnight and the next morning you autoclave them):

	PBS	H_2O
	0.5M EDTA	3M CH_3COONa

Solutions to make with 0.1% DEPC water (you have treated the H20 with DEPC and then you use it for making the solutions):

		70% ethanol
	4M LiCl	Hydrolysis mix
	Heparin	10mg/ml yeast RNA
	10% CHAPS	10% Tween 20

Solutions to keep specially for RNA work:
100% ethanol

Fixatives

For optimal fixation it may be critical to use fresh formaldehyde solutions. Fresh 4% solutions can be made from 16%, methanol free, formaldehyde or from solid paraformaldehyde (4% w/v).

MEMFA Fix: 1 part 10x MEMFA salts + 1 part 37% formaldehyde + 8 parts water

10x MEMFA Salts: 1 M MOPS + 20mM EGTA + 10mM $MgSO_4$ (10x salts can be autoclaved and stored. MEMFA turns yellow after autoclaving).

For *in situ*: fix for 2 hours or O/N @ 4ºc.

4% Paraformaldehyde (PFA) Fixative

1. Place 450 ml of dH_2O in a glass beaker. Heat to 60°C using a hot plate with stirring.

2. While stirring, add 20 g of paraformaldehyde powder to the heated water. Cover and maintain at 60°C.

3. Add 5 drops of 2N NaOH (1 drop per 100 ml). The solution should clear within a couple of minutes (There will be some fine particles that will not go away). **Do not heat solution above 70°C.** PFA will break down at temperatures above 70°C.

4. Remove from heat and add 50 ml of 10X PBS. Adjust pH to 7.2; you may have to add some HCl. Final volume will be 500 ml. Filter and place on ice. Cover with foil to protect from the light.

5. Use immediately, or aliquots may be frozen at -20°C and thawed as needed.

How to prepare TEA - Acetic anhydride?

The TEA (Triethanolamine) molecular weight is FW 149.2

Therefore to prepare 50 mls of 1M TEA, one should weight:
0.05 **l** x 1 **mol.l^-1** x 149.2 **g.mol^-1** = 7.46 **g**

TEA is a liquid. But because it is extremely viscous, there is no way we can measure the volume corresponding to 7.46 g. Rather, it is much more convenient to place a 50 ml FALCON tube on the balance, calibrate to zero, and **transfer 7.46 g with a pipette**.

Then, add DEPC-treated nanopure water to 30 ml, mix well. Fill the tube to 50 ml, mix.

Preparing [TEA – Acetic anhydride] for *in situ* hybridization (0.1M TEA and 0.25% acetic acid):

For a 50 ml tube:
-5ml of TEA 1M
-45 ml of DEPC-treated nanopure water
-Adjust pH to 7 with "pH paper": (add approximately 300 µl of HCl 37%, work under the fume hood)
-Add Acetic anhydride (125 µl)

Shake well and use immediately!

Bench table – Day 1

T°C	Time		Solution															
60°C	1 h.		Dewaxing															
	5’		Cool the slides															
	5’	2x	Xylene															
	5’		EtOH 100%															
	5’		95% EtOH in PBS															
	5’		75% EtOH in PBS															
RT	5’		50% EtOH in PBS															
	5’		25% EtOH in PBS															
	5’	2x	PBS															
	20’		PFA4%-PBS															
	5’	2x	PBS-Tween 0.1%															
37°C	30’		PK 10 µg/ml															
	5’		PFA4%-PBS															
	5’	2x	PBS-Tween0.1%															
	5’		TEA + anhídrido acético															
RT	5’	2x	PBS-Tween 0.1%															
	10’’		PBS 1X															
60°C	2-3 h		Prehybridization buffer															
80°C	5’		Probe denaturation															
4°C	5’		Chill on ice															
60°C	O/N		Hybridization															

Bench table – Day 2

T°C	Time		Solution																
60°C	10’’		4 x SSC<br>0.1% CHAPS																
	30’		2 x SSC<br>0.1% CHAPS/ 50% formamide																
37°C	10’		TNE																
	30’		TNE+RNase A (20ug/mL)																
	10’		TNE																
65°C	20’		SSC2X<br>0.1% CHAPS																
	20’	2X	SSC 0.2X<br>0.1% CHAPS																
RT	5’		50% Buffer1 / 50%<br>[0.2x SSC – 0.1% CHAPS]																
	5’		Buffer 1																
	1hr.		Buffer 1 + 20% serum																
4°C	O/N		anti-DIG-AP 1:2000																

Bench table – Day 3

T°C	Time		Solution																
RT	10’	4x	Buffer 1 + Tween0.05																
	5’	2x	Phosphatase alkaline buffer																
			NBT/BCIP																
	10’	3x	PBS																
	1hr.		MEMFA																
	5’	2X	EtOH 100%																
	5’		Xylene I																
	5’		Carbol-Xylene																
	5’		Xylene II																
	5’		Xylene III																
	5’		Xylene IV																
Mount in Entellan																			
