# Supplementary material for: Molecular footprinting of skeletal tissues in the catshark Scyliorhinus canicula and the clawed frog Xenopus tropicalis identifies conserved and derived features of vertebrate calcification
Source: Front Genet. 2015 Sep 15;6:283. doi: 10.3389/fgene.2015.00283 (PMC4584932; doi:10.3389/fgene.2015.00283)
Supplement: Data Sheet 4 — Molecular Phylogenetic analysis of gnathostome Clade A fibrillar collagen genes. Phylogenetic relationships were inferred and the tree with the highest log likelihood (-7413.8950) is shown. The percentages of trees in which the associated taxa clustered together are indicated. The tree is drawn to scale, and branch lengths correspond to the number of substitutions per site. Orthology groups (OG) are identified as blue circles, and S.c. sequences are shown in red. The tree was rooted according to (Zhang and Cohn, 2008). [file DataSheet4.PDF]

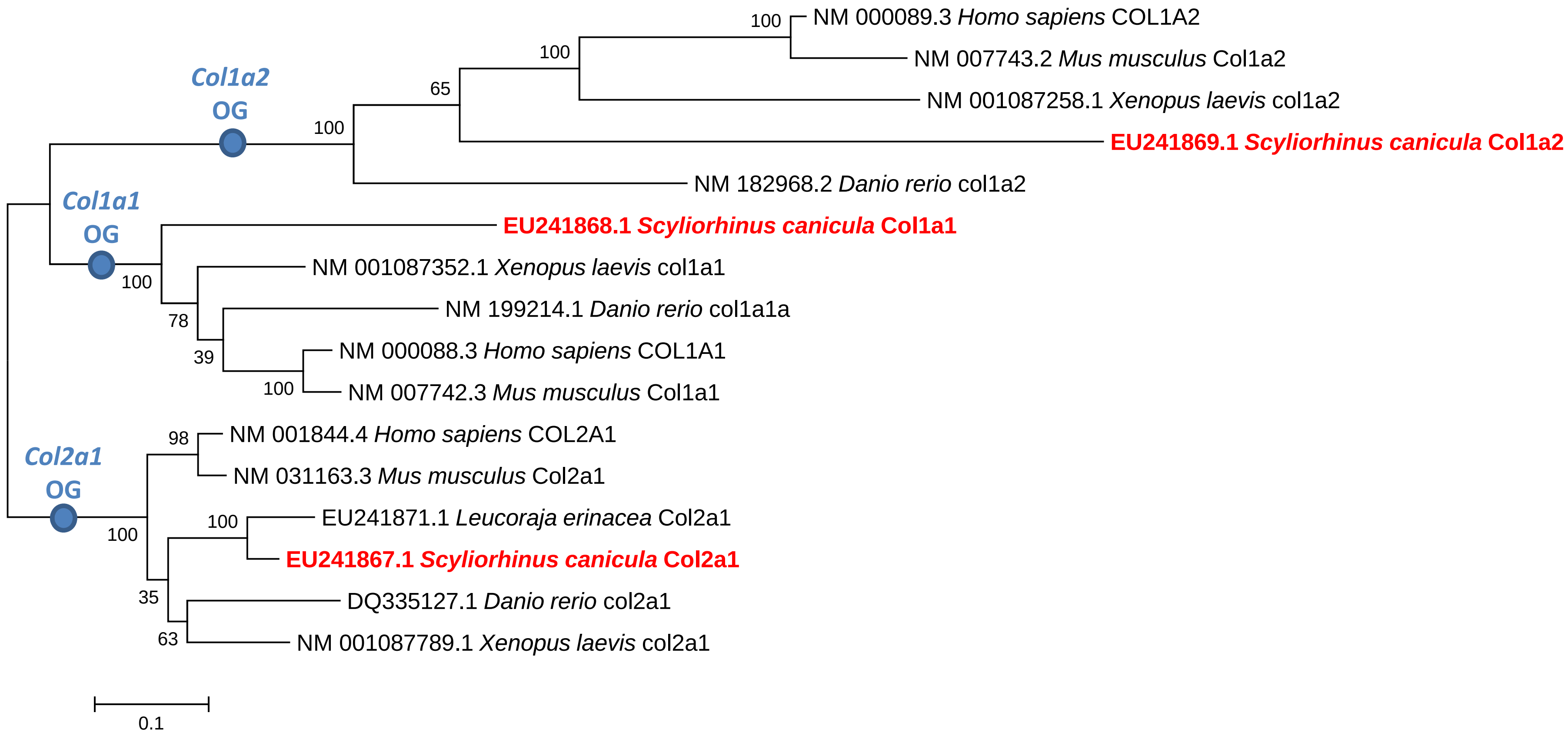


**Supplementary Data 1:** Molecular Phylogenetic analysis of gnathostome Clade A fibrillar collagen genes. Phylogenetic relationships were inferred and the tree with the highest log likelihood (-7413.8950) is shown. The percentages of trees in which the associated taxa clustered together are indicated. The tree is drawn to scale, and branch lengths correspond to the number of substitutions per site. Orthology groups (OG) are identified as blue circles, and catshark sequences are shown in red. The tree was rooted according to Zhang and Cohn 2008.
