# Supplementary material for: Molecular footprinting of skeletal tissues in the catshark Scyliorhinus canicula and the clawed frog Xenopus tropicalis identifies conserved and derived features of vertebrate calcification
Source: Front Genet. 2015 Sep 15;6:283. doi: 10.3389/fgene.2015.00283 (PMC4584932; doi:10.3389/fgene.2015.00283)
Supplement: Data Sheet 5 — Fibrillar collagen sequences. [file DataSheet5.DOCX]

#mega

!Title hop;

!Format DataType=Protein indel=-;

#gi|165881923|gb|EU241869.1|_Scyliorhinus_canicula_Col1a2_(Col1a2)_mRNA_partial_cds

MPGERGAAGSIGIKGEKGDSGPAGPQGSAGMDGPRGIPGPVGAPGPSGPSGDRGESGASG

HSGSAGPRGSPGERGEAGRIGPPGFAGPPGADGHSGAKGEKGVPGGKGEVGSAGAVGQPG

LAGAAGPAGVPGLQGPVGSLGQTGMTGFSGPAGRAGTPGPAGATGPQGPPGPTGNPGPQG

PRGDTGPQGRSGSGGSLGANGVPGDRGPAGETGPSGPPGGPGPQGFLGSAGVHGLPGSRG

DSGLPGVSGPHGLPGPTGPSGASGLRGSAGAVGPAGLTGQNGESGRDGNPGRDGPPGPGG

QSGVKGERGDAGRVGSSGALGQPGAHGHVGPAGKAGNRGEAGPSGSSGAVGQMGPRGLAG

PQGPRGGKGETGFAGVRGLKGLRGPGGLQGLPGVGGISGETGSHGPSGPSGPRGPSGPSG

PHGKDGGSGHPGSIGPVGHRGRPGEHGPMGPPGSPGPAGLPGMAGVPYEVSREKDIEVDA

KLKVLTNQIENIRTPEGSKKNPARSCRDLRLSHPEWKSGYYWIDPNQGCIMDAIRVYCDF

VSGE

#gi|165881927|gb|EU241871.1|_Leucoraja_erinacea_Col2a1_(Col2a1)_mRNA_partial_cds

MPGERGTAGIPGPKGDRGDSGGKGPEGAPGKDGGRGLTGPIGPPGPSSPNGLKGETGPRG

PTGTAGARGAPGERGETGPPGPAGFAGPPGSDGQAGQKGEPGETGQKGDAGAPGPQGPSG

APGPQGPTGVTGPKGARGAQGPPGATGFPGAAGRVGPPGPNGNPGPVGPAGAPGKDGPKG

LRGDPGQAGRAGDPGLQGPAGTPGEKGEPGEDGPPGPDGPSGPQGLAGQRGIVGLPGQRG

ERGFPGLPGPSGEPGKQGAPGSAGDRGPPGPVGPSGLTGPSGEPGREGNPGADGPPGRDG

STGPKGERGQTGVAGAPGASGAPGAPGPVGPTGKQGNRGESGAQGLMGPSGPPGLQGMPG

PQGPRGDKGEAGEAGERGQKGHRGFTGLQGLPGPPGPGGDQGAVGAAGPSGPRGPPGPVG

PSGKDGSNGMPGPIGPPGPRGRSGESGPSGPPGNSGPPGPPGPPGPGIDMSRQHDAEVDA

TLKSLNNQIENIRSPEGSKKNPARTCRDLQLCHSDWKSGDYWVDPNQGCTLDAIKVFCNM

ETGE

#gi|110349771|ref|NM_000088.3|_Homo_sapiens_collagen_type_I_alpha_1_(COL1A1)_mRNA

MPGERGAAGLPGPKGDRGDAGPKGADGSPGKDGVRGLTGPIGPPGPAGAPGDKGESGPSG

PAGPTGARGAPGDRGEPGPPGPAGFAGPPGADGQPGAKGEPGDAGAKGDAGPPGPAGPAG

PPGPIGNVGAPGAKGARGSAGPPGATGFPGAAGRVGPPGPSGNAGPPGPPGPAGKEGGKG

PRGETGPAGRPGEVGPPGPPGPAGEKGSPGADGPAGAPGTPGPQGIAGQRGVVGLPGQRG

ERGFPGLPGPSGEPGKQGPSGASGERGPPGPMGPPGLAGPPGESGREGAPGAEGSPGRDG

SPGAKGDRGETGPAGPPGAPGAPGAPGPVGPAGKSGDRGETGPAGPAGPVGPVGARGPAG

PQGPRGDKGETGEQGDRGIKGHRGFSGLQGPPGPPGSPGEQGPSGASGPAGPRGPPGSAG

APGKDGLNGLPGPIGPPGPRGRTGDAGPVGPPGPPGPPGPPGPPSAGFDFSRDRDLEVDT

TLKSLSQQIENIRSPEGSRKNPARTCRDLKMCHSDWKSGEYWIDPNQGCNLDAIKVFCNM

ETGE

#gi|48762933|ref|NM_000089.3|_Homo_sapiens_collagen_type_I_alpha_2_(COL1A2)_mRNA

LPGERGAAGIPGGKGEKGEPGLRGEIGNPGRDGARGAPGAVGAPGPAGATGDRGEAGAAG

PAGPAGPRGSPGERGEVGPAGPNGFAGPAGAAGQPGAKGERGAKGPKGENGVVGPTGPVG

AAGPAGPNGPPGPAGSRGDGGPPGMTGFPGAAGRTGPPGPSGISGPPGPPGPAGKEGLRG

PRGDQGPVGRTGEVGAVGPPGFAGEKGPSGEAGTAGPPGTPGPQGLLGAPGILGLPGSRG

ERGLPGVAGAVGEPGPLGIAGPPGARGPPGAVGSPGVNGAPGEAGRDGNPGNDGPPGRDG

QPGHKGERGYPGNIGPVGAAGAPGPHGPVGPAGKHGNRGETGPSGPVGPAGAVGPRGPSG

PQGIRGDKGEPGEKGPRGLPGLKGHNGLQGLPGIAGHHGDQGAPGSVGPAGPRGPAGPSG

PAGKDGRTGHPGTVGPAGIRGPQGHQGPAGPPGPPGPPGPPGVSGGGYDFGRPKDYEVDA

TLKSLNNQIETLLTPEGSRKNPARTCRDLRLSHPEWSSGYYWIDPNQGCTMDAIKVYCDF

STGE

#gi|111118975|ref|NM_001844.4|_Homo_sapiens_collagen_type_II_alpha_1_(COL2A1)_transcript_variant_1_mRNA

MPGERGAAGIAGPKGDRGDVGEKGPEGAPGKDGGRGLTGPIGPPGPAGANGEKGEVGPPG

PAGSAGARGAPGERGETGPPGPAGFAGPPGADGQPGAKGEQGEAGQKGDAGAPGPQGPSG

APGPQGPTGVTGPKGARGAQGPPGATGFPGAAGRVGPPGSNGNPGPPGPPGPSGKDGPKG

ARGDSGPPGRAGEPGLQGPAGPPGEKGEPGDDGPSGAEGPPGPQGLAGQRGIVGLPGQRG

ERGFPGLPGPSGEPGKQGAPGASGDRGPPGPVGPPGLTGPAGEPGREGSPGADGPPGRDG

AAGVKGDRGETGAVGAPGAPGPPGSPGPAGPTGKQGDRGEAGAQGPMGPSGPAGARGIQG

PQGPRGDKGEAGEPGERGLKGHRGFTGLQGLPGPPGPSGDQGASGPAGPSGPRGPPGPVG

PSGKDGANGIPGPIGPPGPRGRSGETGPAGPPGNPGPPGPPGPPGPGIDMSRQHDAEVDA

TLKSLNNQIESIRSPEGSRKNPARTCRDLKLCHPEWKSGDYWIDPNQGCTLDAMKVFCNM

ETGE

#gi|56790314|ref|NM_199214.1|_Danio_rerio_collagen_type_I_alpha_1a_(col1a1a)_mRNA

MPGERGAAGLPGLKGDRGDQGAKGADGAAGKDGIRGMTGPIGPPGPAGAPGDKGESGAQG

LVGPTGARGPPGERGETGAPGPAGFAGPPGADGLPGAKGEPGDNGAKGDAGAPGPAGATG

APGPQGPVGATGPKGARGAAGPPGATGFPGAAGRVGPPGPSGNSGPPGPPGPAGKEGQKG

NRGETGPAGRTGEVGAAGPPGAPGEKGNPGAEGATGPAGIPGPQGIGGQRGIVGLPGQRG

ERGFPGLPGPSGEIGKQGPSGPSGERGPPGPMGPPGLAGPPGEPGREGTPGNEGSAGRDG

AAGPKGDRGETGPSGTPGAPGPPGAAGPIGPAGKTGDRGETGPAGVPGPAGPSGPRGPSG

PAGARGDKGETGEAGERGMKGHRGFTGMPGPPGPPGPSGESGPAGASGPAGPRGPAGSAG

SAGKDGMSGLPGPIGPPGPRGRNGEIGPAGPPGPPGPPGAPGPSGGGFDIGRDRDLEVDT

TLKSLSQQIESIISPDGTKKNPARTCRDLKMCHPDWKSGEYWIDPDQGCNQDAIKVYCNM

ETGE

#gi|48762666|ref|NM_182968.2|_Danio_rerio_collagen_type_I_alpha_2_(col1a2)_mRNA

MPGERGAAGTPGAKGEKGEAGYRGLEGNAGKDGARGAPGPSGPPGPAGANGDKGETGSFG

PPGPAGPRGAPGERGESGPAGPSGFAGPPGADGQTGPRGEKGPAGGKGDAGPAGPAGPAG

NTGPLGPSGPVGPPGARGDSGPTGLTGFPGAPGRVGPPGPAGIVGPAGLTGPAGKDGPRG

PRGDVGPAGPPGENGMIGPLGLAGEKGPPGEAGAPGAPGPAGPQGQLGSQGFNGLPGSRG

DRGLPGIPGSVGEPGRVGPAGAPGARGPGGNIGMPGMTGPQGEAGREGSPGNDGPPGRPG

AAGIKGDRGEPGSPGTAGPVGAPGPNGPSGAVGRPGNRGESGPSGPTGAVGPAGARGAPG

PAGPRGEKGVAGEKGDRGMKGLRGHPGLQGMPGPNGPSGDSGPAGIAGPSGPRGPAGPNG

PAGKDGSNGMPGAIGPPGHRGPAGHVGPAGPPGSPGLPGPPGPSGGGYDTSRAKDYEVDA

TIKSLNTQIENLLSPEGSKKNPARTCRDIRLSHPEWSSGFYWIDPNQGCTMDAIKAFCDF

STGQ

#gi|84626355|gb|DQ335127.1|_Danio_rerio_collagen_type_II_alpha_1_(col2a1)_mRNA_complete_cds

MPGERGAVGISGAKGDRGDSGEKGPEGAPGKDGSRGLTGPIGPPGPSGPNGAKGETGPIG

SIGAPGARGAPGDRGEIGAPGPAGFAGPPGADGQPGNKGEQGESGQKGDSGAPGPQGPSG

APGPVGPTGVTGPKGARGAQGAPGATGFPGAAGRVGPPGPNGNPGAAGPAGPSGKDGPKG

VRGDAGPPGRAGDAGLRGPPGAPGEKGEAGEDGPPGPDGPSGPAGLAGQRGIVGLPGQRG

ERGFPGLPGPSGEPGKQGAPGGSGDRGPPGPVGPPGLTGPAGETGREGNPGSDGPPGRDG

AAGVKGERGNTGPIGAPGAPGAPGAPGSVGPIGKQGDRGENGPQGPAGPPGPAGARGMVG

PQGPRGDKGEAGEAGERGQKGHRGFTGLQGLPGPPGSPGDQGAAGPAGPSGAKGPSGPVG

PAGKDGSNGQPGPIGPPGPRGRSGESGPVGPPGNPGPPGPPGPPGPGIDMSRQHDVEVDA

TLKSINGQIEDIRSPDGSRKNPARSCRDLKLCHPEWKSGDYWVDPNLGSAADAIKVFCNM

ETGE

#gi|148222552|ref|NM_001087352.1|_Xenopus_laevis_collagen_type_I_alpha_1_(col1a1)_mRNA

MPGERGSSGLPGAKGDRGDQGVKGSDGTPGKDGVRGLTGPIGPPGPGGAPGDKGEAGPAG

PAGPTGARGAPGERGESGPPGPAGFAGPPGADGQPGAKGEQGDSGAKGDAGPPGPAGPTG

APGPAGALGSPGPKGARGAPGPPGATGFPGAAGRLGPPGPSGNAGPPGPSGPAGKEGAKG

PRGETGPAGRSGEPGAAGPPGPPGEKGSPGSDGPAGAPGIPGPQGVAGSRGTVGLPGMRG

ERGFSGLPGPAGEPGKQGPSGPSGERGPPGPSGPPGLGGPPGESGREGAPGSEGAPGRDG

AVGPKGDRGEGGPAGPPGAPGAPGAPGPVGPAGKSGDRGETGPSGPAGPAGTAGARGPAG

PQGPRGDKGEAGEQGERGMKGHRGFNGPSGPPGPPGSSGEQGPSGASGPAGPRGPPGSSG

NPGKDGSNGLPGPIGPPGPRGRTGDVGPAGPPGPPGPPGPPGQSGGGFDFSRDRDLEVDS

TLKSLSKQIENIRSPEGTRKNPARTCRDLKMCHSDWKSGEYWIDPNQGCILDAIKVYCNM

ETGE

#gi|147898762|ref|NM_001087258.1|_Xenopus_laevis_collagen_type_1_alpha_2_(col1a2)_mRNA

IPGERGAAGVPGSKGEKGDAGHAGEYGNQGRDGSRGPAGASGAPGPAGAAGDRGESGPAG

PAGIAGPRGTPGERGEAGPAGTTGFAGPPGAAGHTGVKGERGPKGPKGEGGSPGALGAVG

SHGPAGPNGPAGTTGPRGDGGAPGATGFPGPAGRTGAPGPAGTVGPSGPTGHPGKDGPRG

TRGDSGPVGRPGEQGIGGPQGLSGEKGPSGESGPAGAPGASGPSGVLGSLGFSGLPGSRG

ERGTPGGSGSNGEPGPSGPPGAAGARGPSGPMGSPGPNGVPGEAGRDGNPGNDGPSGRDG

LPGNKGERGYPGNSGPAGSLGASGAPGAVGPAGKSGNRGEPGPVGPAGVVGPAGPRGPAG

IQGGRGDKGEAGEKGARGLDGRKGHNGLQGLPGPAGSPGETGPAGTNGPSGPRGPAGPSG

PPGKEGRSGHSGTIGPVGLRGPAGHQGPAGPPGPPGLPGLPGSSGGGYDGGRPKDYEVDA

TLKSLNSQIETIMTPEGSKKNPARTCRDLRLSHPDWSSGFYWIDPNQGCTSDAIRVFCDF

STGE

#gi|148233277|ref|NM_001087789.1|_Xenopus_laevis_collagen_type_II_alpha_1_(col2a1)_mRNA

MPGERGAAGISGPKGDRGDTGEKGPEGASGKDGSRGLTGPIGPPGPAGPNGEKGESGPSG

PPGIVGARGAPGDRGENGPPGPAGFAGPPGADGQSGLKGDQGESGQKGDAGAPGPQGPSG

APGPQGPTGVFGPKGARGAQGPAGATGFPGAAGRVGTPGPNGNPGPPGPPGSAGKEGPKG

VRGDAGPPGRAGDPGLQGAAGAPGEKGEPGEDGPSGPDGPPGPQGLSGQRGIVGLPGQRG

ERGFPGLPGPSGEPGKQGGPGSSGDRGPPGPVGPPGLTGPSGEPGREGNPGSDGPPGRDG

ATGIKGDRGETGPLGAPGAPGAPGAPGSVGPTGKQGDRGESGPQGPLGPSGPAGARGLAG

PQGPRGDKGEAGEAGERGQKGHRGFTGLQGLPGPPGSAGDQGATGPAGPAGPRGPPGPVG

PSGKDGSNGISGPIGPPGPRGRSGETGPSGPPGQPGPPGPPGPPGPGIDMSNSLPVDVEA

TLKSLNNQIENIRSPDGTKKNPARTCRDLKLCHPEWKSGDYWIDPNQGCTVDAIKVFCDM

ETGE

#gi|165881921|gb|EU241868.1|_Scyliorhinus_canicula_Col1a1_(Col1a1)_mRNA_partial_cds

MPGDRGMSGLPGAKGVRGESGPKGGDGAPGKDGGRGMTGAIGPPGHSGAPGEKGEAGPPG

PSGPTGGRGAPGERGESGSPGPAGFAGPPGADGQPGAKGEVGDSGPKGDHGPQGAIGAVG

IAGPTGPAGPPGSKGARGAPGSPGATGFPGAAGRVGPPGPSGNTGPSAPAGAVGKEGAKG

IRGETGSAGRPGEPGNVGAPGPAGEKGSPGSDGPPGASGIPGPQGITGMRGIVGMSGVRG

ERGSMGLSGKTGEPGKAGPVGAPGDRGPPGPMGPPGLAGPSGESGREGAPGAEGAPGRDG

ASGPKGDRGEPGTSGIPGAPGAPGAPGPVGPSGKNGDRGEAGPSGPAGPSGPMGPRGPLG

AVGPRGDRGESGEAGLRGIKGHRGFTGMQGLPGPTGPPGEQGPAGSSGASGPRGPPGPSG

SPGKDGASGLPGPIGPPGPRGRNGDMGPAGPPGAPGVPGPPGPSSGGFDFQGDRDIQIDT

TLKSLTQQIENIRSPEGTRKNPARTCRDLKMCHPEWKSGDYWIDPNQGCTLDAIRVYCNL

ETGE

#gi|165881919|gb|EU241867.1|_Scyliorhinus_canicula_Col2a1_(Col2a1)_mRNA_partial_cds

MPGERGTAGIPGPKGDRGDNGEKGPEGAPGKDGGRGLTGPIGPPGPAGPNGEKGESGPRG

PTGAAGSRGAPGERGETGPPGPAGFAGPPGSDGQAGAKGEAGETGQKGDAGAPGPQGPSG

APGPQGPTGVTGPKGARGAQGPPGATGFPGAAGRVGPPGPNGNPGPVGPPGSPGKDGPKG

LRGDPGQAGRAGDPGLQGPAGASGEKGEPGEDGPPGPDGPSGPQGLAGQRGIVGLPGQRG

ERGFPGLPGPSGEPGKQGAPGSAGDRGPPGPVGPPGLTGPSGEPGREGNPGADGPPGRDG

AAGMKGDRGQTGPAGAPGSPGGPGAPGPVGPTGKQGNRGEPGAQGPMGPSGPAGARGMPG

PQGPRGDKGEAGETGERGQKGHRGFTGLQGLPGPPGPSGDQGAVGAAGPAGPRGPPGPVG

PSGKDGANGLTGPIGPPGPRGRSGETGPSGPPGNSGPPGPPGPPGPGIDMSRQHDAEVDA

TLKSLNNQIENIRSPEGSKKNPTRTCRDLKLCHSDWKSGDYWIDPNQGCTLDAIKVFCNM

ETGE

#gi|118131144|ref|NM_007742.3|_Mus_musculus_collagen_type_I_alpha_1_(Col1a1)_mRNA

MPGERGAAGLPGPKGDRGDAGPKGADGSPGKDGARGLTGPIGPPGPAGAPGDKGEAGPSG

PPGPTGARGAPGDRGEAGPPGPAGFAGPPGADGQPGAKGEPGDTGVKGDAGPPGPAGPAG

PPGPIGNVGAPGPKGPRGAAGPPGATGFPGAAGRVGPPGPSGNAGPPGPPGPVGKEGGKG

PRGETGPAGRPGEVGPPGPPGPAGEKGSPGADGPAGSPGTPGPQGIAGQRGVVGLPGQRG

ERGFPGLPGPSGEPGKQGPSGSSGERGPPGPMGPPGLAGPPGESGREGSPGAEGSPGRDG

APGAKGDRGETGPAGPPGAPGAPGAPGPVGPAGKNGDRGETGPAGPAGPIGPAGARGPAG

PQGPRGDKGETGEQGDRGIKGHRGFSGLQGPPGSPGSPGEQGPSGASGPAGPRGPPGSAG

SPGKDGLNGLPGPIGPPGPRGRTGDSGPAGPPGPPGPPGPPGPPSGGYDFSRDRDLEVDT

TLKSLSQQIENIRSPEGSRKNPARTCRDLKMCHSDWKSGEYWIDPNQGCNLDAIKVYCNM

ETGQ

#gi|111120328|ref|NM_007743.2|_Mus_musculus_collagen_type_I_alpha_2_(Col1a2)_mRNA

LPGERGAAGIPGGKGEKGETGLRGDTGNTGRDGARGIPGAVGAPGPAGASGDRGEAGAAG

PSGPAGPRGSPGERGEVGPAGPNGFAGPAGAAGQPGAKGEKGTKGPKGENGIVGPTGSVG

AAGPSGPNGPPGPVGSRGDGGPPGMTGFPGAAGRTGPPGPSGIAGPPGPPGAAGKEGIRG

PRGDQGPVGRTGETGASGPPGFVGEKGPSGEPGTAGAPGTAGPQGLLGAPGILGLPGSRG

ERGLPGIAGALGEPGPLGISGPPGARGPPGAVGSPGVNGAPGEAGRDGNPGSDGPPGRDG

QPGHKGERGYPGSIGPTGAAGAPGPHGSVGPAGKHGNRGEPGPAGSVGPVGAVGPRGPSG

PQGIRGDKGEPGDKGHRGLPGLKGYSGLQGLPGLAGLHGDQGAPGPVGPAGPRGPAGPSG

PVGKDGRSGQPGPVGPAGVRGSQGSQGPAGPPGPPGPPGPPGVSGGGYDFGRPKDYEVDA

TLKSLNNQIETLLTPEGSRKNPARTCRDLRLSHPEWNSDYYWIDPNQGCTMDAIKVYCDF

STGE

#gi|166064039|ref|NM_031163.3|_Mus_musculus_collagen_type_II_alpha_1_(Col2a1)_transcript_variant_1_mRNA

MPGERGAAGIAGPKGDRGDVGEKGPEGAPGKDGGRGLTGPIGPPGPAGANGEKGEVGPPG

PSGSTGARGAPGERGETGPPGPAGFAGPPGADGQPGAKGDQGEAGQKGDAGAPGPQGPSG

APGPQGPTGVTGPKGARGAQGPPGATGFPGAAGRVGPPGANGNPGPAGPPGPAGKDGPKG

VRGDSGPPGRAGDPGLQGPAGAPGEKGEPGDDGPSGLDGPPGPQGLAGQRGIVGLPGQRG

ERGFPGLPGPSGEPGKQGAPGASGDRGPPGPVGPPGLTGPAGEPGREGSPGADGPPGRDG

AAGVKGDRGETGALGAPGAPGPPGSPGPAGPTGKQGDRGEAGAQGPMGPSGPAGARGIAG

PQGPRGDKGESGEQGERGLKGHRGFTGLQGLPGPPGPSGDQGASGPAGPSGPRGPPGPVG

PSGKDGSNGIPGPIGPPGPRGRSGETGPVGPPGSPGPPGPPGPPGPGIDMSRQHDVEVDA

TLKSLNNQIESIRSPDGSRKNPARTCQDLKLCHPEWKSGDYWIDPNQGCTLDAMKVFCNM

ETGE
